# Human Milk Oligosaccharides Modulate the Risk for Preterm Birth in a Microbiome-Dependent and -Independent Manner

**DOI:** 10.1128/mSystems.00334-20

**Published:** 2020-06-09

**Authors:** Manuela-Raluca Pausan, Vassiliki Kolovetsiou-Kreiner, Gesa Lucia Richter, Tobias Madl, Elisabeth Giselbrecht, Barbara Obermayer-Pietsch, Eva-Christine Weiss, Evelyn Jantscher-Krenn, Christine Moissl-Eichinger

**Affiliations:** a Interactive Microbiome Research, Department of Internal Medicine, Medical University of Graz, Graz, Austria; b Department of Obstetrics and Gynecology, Medical University of Graz, Graz, Austria; c Gottfried Schatz Research Center for Cell Signaling, Metabolism and Aging, Division of Molecular Biology and Biochemistry, Medical University of Graz, Graz, Austria; d Department of Internal Medicine, Division of Endocrinology and Diabetology and Department of Obstetrics and Gynecology, Endocrinology Lab Platform, Medical University of Graz, Graz, Austria; e BioTechMed, Graz, Austria; Pacific Northwest National Laboratory

**Keywords:** pregnancy, preterm delivery, vaginal and urinary microbiome, human milk oligosaccharides, inflammation, secretor status, 3′-sialyllactose, 3′SL, metabolites, preterm birth, tocolysis, urinary microbiome, vaginal microbiome

## Abstract

Preterm birth (PTB) is one of the leading causes of neonatal mortality. The causes for spontaneous PTB are multifactorial and often remain unknown. In this study, we tested the hypothesis that human milk oligosaccharides (HMOs) in blood and urine modulate the maternal urinary and vaginal microbiome and influence the risk for PTB. We analyzed the vaginal and urinary microbiome of a cross-sectional cohort of women with or without preterm labor and correlated our findings with measurements of metabolites and HMOs in urine and blood. We identified several microbial signatures, such as Lactobacillus jensenii, L. gasseri, *Ureaplasma* sp., and *Gardnerella* sp., associated with a short cervix, PTB, and/or preterm contractions. In addition, we observed associations between sialylated HMOs, in particular 3′-sialyllactose, with PTB, short cervix, and increased inflammation and confirmed an influence of HMOs on the microbiome profile. Since they identify serum and urinary HMOs and several key microorganisms associated with PTB, our findings point at two distinct processes modulating the risk for PTB. One process seems to be driven by sterile inflammation, characterized by increased concentrations of sialylated HMOs in serum. Another process might be microbiome mediated and potentially associated with specific HMO signatures in urine. Our results support current efforts to improve diagnostics and therapeutic strategies in PTB.

**IMPORTANCE** The causes for preterm birth (PTB) often remain elusive. We investigated whether circulating human milk oligosaccharides (HMOs) might be involved in modulating urinary and vaginal microbiome promoting or preventing PTB. We identified here HMOs and key microbial taxa associated with indicators of PTB. Based on our results, we propose two models for how HMOs might modulate risk for PTB: (i) by changes in HMOs associated with sterile inflammation (microbiome-independent) and (ii) by HMO-driven shifts in microbiome (microbiome-dependent). Our findings will guide current efforts to better predict the risk for PTB in seemingly healthy pregnant women and also provide appropriate preventive strategies.

## Abstract (precis)

The causes for preterm birth (PTB) often remain elusive. We investigated whether circulating human milk oligosaccharides (HMOs) might be involved in modulating urinary and vaginal microbiome promoting or preventing PTB. We identified here HMOs and key microbial taxa associated with indicators of PTB. Based on our results, we propose two models for how HMOs might modulate risk for PTB: (i) by changes in HMOs associated with sterile inflammation (microbiome-independent) and (ii) by HMO-driven shifts in microbiome (microbiome-dependent). Our findings will guide current efforts to better predict the risk for PTB in seemingly healthy pregnant women and also provide appropriate preventive strategies.

## INTRODUCTION

Each year, approximately 15 million infants worldwide are born preterm (prior to the week 37 of gestation [1]). With rates ranging from 5 to 18% of all births across 184 countries (Europe, 6% [2, 3]), preterm birth (PTB) is the leading cause of mortality in children under the age of 5 years (3, 4). Besides the emotional burden on affected families, PTB also has severe economic consequences for society.

Spontaneous PTB is a multifactorial process, often accompanied by infections, inflammation, uteroplacental ischemia, stress, or other immunologically mediated processes (5). Risk factors include certain lifestyle habits (e.g., smoking, alcohol and drug abuse, and stress), demographic factors (maternal age over 35 or under 17, ethnicity, and low socioeconomic status), medical conditions (infections, diabetes, high blood pressure, obesity, multiple pregnancies, and immunological complications), and genetic predisposition (6). However, in up to half of all cases, the causes remain unknown (7).

Recently, differences in maternal microbiomes have been linked to PTB. The vaginal microbiome, which is generally categorized into five community state types (CSTs), undergoes substantial changes over the course of a healthy pregnancy (8), leading to enrichment of specific *Lactobacillus* species (9). Lactobacilli contribute to lowering the vaginal pH by lactic acid production, which provides protection from ascending infections. However, the beneficial role of lactobacilli seems to be based on the species or even the strain level. Both a high abundance of distinct *Lactobacillus* species and a generally low abundance of lactobacilli (along with bacterial vaginosis) have been associated with PTB (10, 11). Perturbation of this well-attuned microbiome, followed by microbial imbalance (dysbiosis) or changes in specific taxa, might lead to infections and PTB (12). In addition, immune factors, such as beta-defensin-2, are thought to modulate the risk for PTB, partly independently of the microbiome (13). To date, a causal link between vaginal microbiome composition and PTB is still lacking.

Other potential risk factors for PTB are urinary tract infections (UTIs), which often remain asymptomatic and are thus overlooked. Although studies linking the urinary microbiome with PTB are rare, certain microbial taxa have been associated with PTB (14).

Human milk oligosaccharides (HMOs) have emerged as potential novel factors involved in maintaining a healthy pregnancy. HMOs are structurally diverse, bioactive glycans in human milk that are already present in the serum (15, 16) and urine of pregnant women (17, 18) as early as 10 weeks of gestation. We have previously shown variations in serum HMO concentration and composition with gestational age (GA) and secretor status (15, 16). Women with a positive secretor status have an active *Secretor* gene encoding α1-2 fucosyltransferase-2 (FUT2) and thus can produce α1-2 fucosylated HMOs. Apart from these genetic factors, metabolic factors were also associated with serum HMOs in pregnancy (15), with as-yet-unknown consequences.

HMOs mediate their various functions either directly, by acting on body cells, or indirectly, by acting on microbial commensals or pathogens. For example, HMOs exert immunomodulatory, anti-inflammatory effects by regulating cytokine expression in macrophages, thereby inducing Th2 polarization (19) or dampening the lipopolysaccharide response (20). HMOs also shape microbial communities, as shown for the gut, serving as substrates for specific strains of genera such as *Bifidobacterium* and *Bacteroides* (21, 22) and *Lactobacillus* (23, 24). In addition to their prebiotic activities toward beneficial microbes, HMOs also directly act on microbes as antiadhesives (25–28) or antimicrobials (29). These findings suggest that HMOs present at particular body sites have direct or indirect effects on microbial communities at these sites. Although the abundance of HMOs in urine has been known for decades (17), the physiological composition or biological roles of urinary HMOs in pregnancy-related disorders such as PTB have not yet been investigated.

We hypothesize here that the maternal vaginal and urinary microbiome plays a critical role in triggering PTB. We consider HMOs in serum and urine to be important regulators of the microbiome, causing subtle changes in certain microbial taxa, preventing or promoting PTB. To test this hypothesis, we performed a cross-sectional study to understand the interplay of the microbiome profile, the urinary metabolome, and serum and urine HMOs in women with preterm labor and thus at risk for spontaneous PTB (*n* = 33) in comparison to appropriate controls (*n* = 27).

## RESULTS

**FIG 1:**
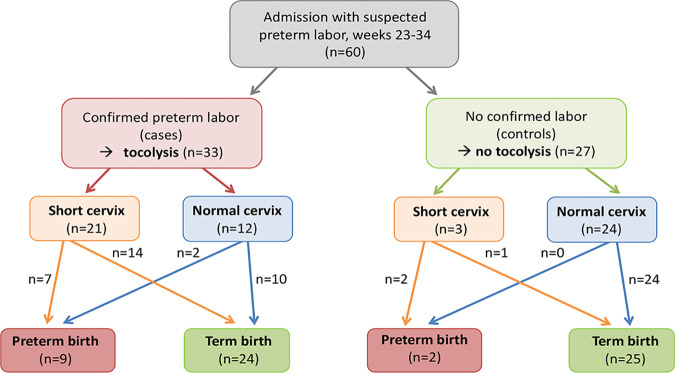
Flow chart of the study population. Based on medical examinations, pregnant women (*n* = 60) were allocated into the case group (confirmed labor, tocolytic treatment; *n* = 33) and the control group (no labor; *n* = 27).

We recruited 60 women with suspected preterm labor, all between weeks 23 and 34 of pregnancy. Before any treatment, a vaginal swab, catheter urine, and blood were collected. Cardiotocography (CTG) examinations were performed to objectively determine uterine contractions, and transvaginal scans were used to measure cervical length. Tocolytics were administered when more than four contractions within 30 min were recorded and clearly felt by the patient. A total of 55% of all women received tocolytics to prevent PTB (“cases”). A total of 40% of all women had a short cervix; the majority of these women received tocolytics, and 18% of all women (27% of the cases) delivered preterm. A total of 82% of the women with subsequent PTB had a short cervix at hospital presentation. Two women did not receive tocolytic treatment for medical reasons despite having preterm labor and delivered preterm at GA 33^+5^ and 33^+6^ (Fig. 1).

Table 1 summarizes sociodemographic and clinical characteristics of case and control group. The two groups did not significantly differ in ethnicity, body mass index (BMI), age, pregnancy hormones, or the inflammatory marker CRP, while there was a trend toward higher leukocyte counts in the cases. The only statistically significant difference at hospital presentation was with respect to cervical length (Table 1). The percentage of women receiving anti-infective treatment in the remainder of their pregnancies was not different between case and control group (42% versus 41%, respectively). The percentage of women with PTB was higher in the tocolysis group without reaching significance (27% versus 7%, *P* = 0.091). When excluding the two women in the control group who had not received tocolysis treatment despite objectively measured contractions, the rate of PTB was significantly different between the groups (chi square, *P* = 0.004). However, no other results changed (not shown). We then analyzed the groups with respect to HMOs (blood and urine), vaginal and urinary microbiome (qualitative), bacterial load (quantitative), and urine metabolites. Metadata used for correlation analyses are provided in Table S1 in the supplemental material.

**TABLE 1:** Group characteristics

Characteristic	No. (%) of subjects^*a*^		*P*
	Tocolysis group	Control group	
Received tocolytics	Yes (*n* = 33)	No (*n* = 27)	
Mean maternal age (yr) ± SEM (range)	29.5 ± 0.9 (20–39)	29.0 ± 1.1 (18–41)	0.746
18–25 yr	7 (21)	6 (22)	
26–35 yr	21 (64)	16 (59)	
36–41 yr	5 (15)	5 (19)	
Cervical length (mm) ± SEM (range)	22.5 ± 1.7 (6–40)	34.9 ± 1.9 (6.8–50)	<0.05
Mean BMI ± SEM (range)	22.3 ± 0.63 (15.4–30.9)	24.3 ± 0.88 (16.9–35.7)	0.055
Wt category (BMI)			
Underweight (<18.5)	5 (15)	4 (15)	
Normal wt (18.5–24.9)	22 (67)	11 (41)	
Overweight (25.0–29.9)	4 (12)	9 (33)	
Obese (>30)	2 (6)	3 (11)	
Ethnicity			0.141
White	32 (97)	23 (85)	
Asian	0 (0)	3 (11)	
Black	1 (3)	1 (4)	
Mean ± SEM (range)			
Gestational age (wks) at sampling	29^+2^ ± 0.63 (23^+3^–33^+4^)	28^+4^ ± 0.6 (23^+4^–33^+1^)	0.659
CRP (ng/ml)	7.43 ± 1.32 (0.9–30.1)	9.76 ± 3.30 (0.8–86.2)	0.841
Leukocyte count (G/liter)	13.77 ± 1.58	10.80 ± 0.63	0.061
Secretor status			
Secretor	29 (88)	21 (78)	0.322
Nonsecretor	4 (12)	6 (22)	
Mean pH ± SEM (range)			
Vaginal	4.85 ± 0.14 (4–7)	4.73 ± 0.16 (3–7)	0.743
Urinary	6.53 ± 0.14 (5.5–8)	6.69 ± 0.31 (5–8)	0.213
Mean concn ± SEM			
Prolactin (ng/ml)	126.4 ± 8.3	123.1 ± 10.2	0.802
Progesterone (ng/ml)	82.6 ± 6.6	78.1 ± 6.6	0.627
Estrogen (E2) (pg/ml)	9,496 ± 521	8,625 ± 744	0.343
Anti-infective treatment	14 (42)	11 (41)	0.895
Delivery time			
Term	24 (73)	25 (93)	0.091
Preterm	9 (27)	2 (7)	

a Values are expressed as the number of subjects (% total) unless stated otherwise in column 1.

10.1128/mSystems.00334-20.9 TABLE S1 All cohort-derived metadata which were used for correlation analysis (A), an RSV table with all detected microbial taxa and their abundance in the respective samples (B), and a list of RSVs, which were detected also in negative controls and thus removed from the dataset (C). This procedure resulted in Table S1B in the supplemental material. Download Table S1, XLSX file, 1.7 MB. Copyright © 2020 Pausan et al. 2020 Pausan et al. This content is distributed under the terms of the Creative Commons Attribution 4.0 International license.

### The HMO signatures in serum and urine are different in women prone to PTB.

**FIG 2:**
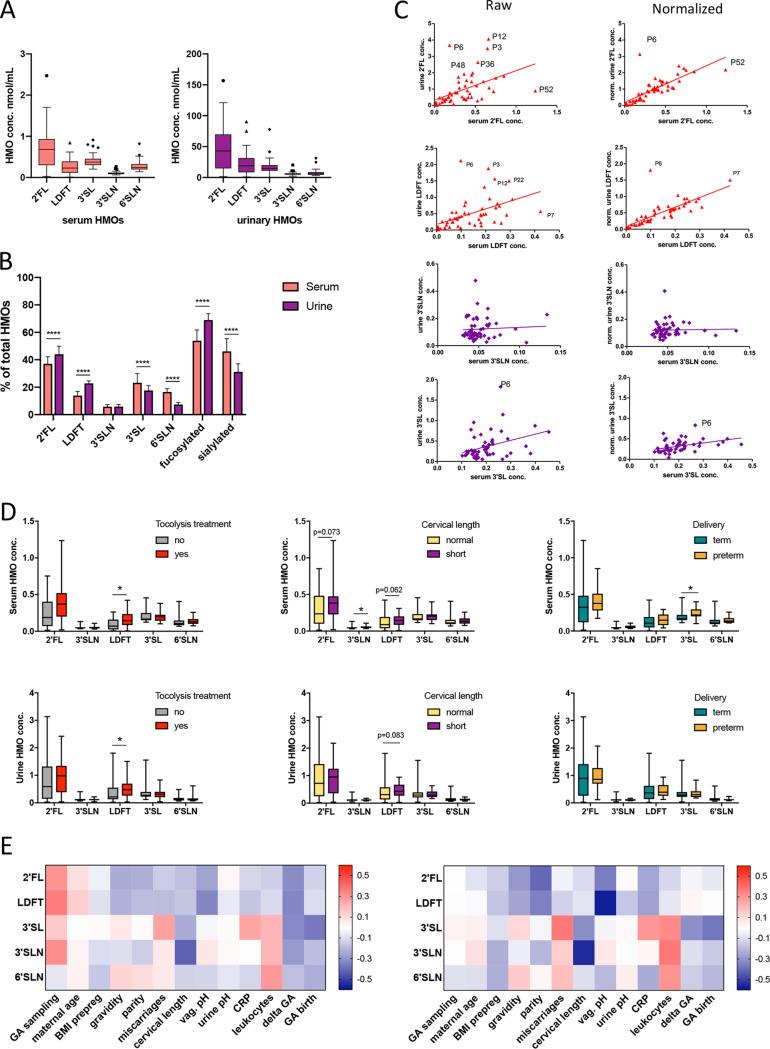
Serum and urinary HMOs are associated with preterm indicators and PTB. (A) Absolute HMOs concentration in serum (red boxes) and in urine (purple boxes). (B) Relative HMO concentrations of serum and urine. (C) Linear regression of serum and urine 2′FL (row 1), LDFT (row 2), 3′SLN (row 3), and 3′SL (row 4) before and after normalization with osmolality. (D) Specific HMO concentrations were different in serum (upper row) and urine (lower row) within the three groups (Mann-Whitney *t* test). LDFT was increased in pregnant women who received tocolytic treatment compared to controls; 3′SLN was higher in women with a short cervix versus a normal cervix, and 3′SL was increased in women with PTB versus term birth. (E) Heatmap of Spearman correlations between serum HMO concentration and maternal variables. The left panel shows correlations with unadjusted HMOs; the right panel shows associations of HMOs after correcting for the gestational age at sampling. GA, gestational age; delta GA, days between sampling and birth; 2′FL, 2′-fucosyllactose; LDFT, lactodifucotetraose; 3′SL, 3′-sialyllactose; 3′SLN, 3′-sialyllacosamine; 6′SLN, 6′-sialyllactosamine; *, *P* < 0.05; **, *P* < 0.01; ***, *P* < 0.001; ****, *P* < 0.0001.

Using high-pressure liquid chromatography (HPLC), we detected HMOs in all maternal serum and urine samples and quantified the five most abundant HMOs. In serum, the median concentrations (95% confidence interval [CI] of the median) were 0.68 nmol/ml (0.41 to 0.81) for 2′-fucosyllactose (2′FL), 0.23 nmol/ml for lactodifucotetraose (LDFT) (0.16 to 0.36), 0.37 nmol/ml (0.32 to 0.41) for 3′-sialyllactose (3′SL), 0.093 nmol/ml (0.087 to 0.10) for 3′-sialyllactosamine (3′SLN), and 0.24 nmol/ml (0.21 to 0.27) for 6′-sialyllactosamine (6′SLN) (Fig. 2A). The same HMO species were present in urine, although in ∼50-fold higher concentrations (Fig. 2A). Urine profiles showed significantly higher proportions of the fucosylated HMOs, 2′FL and LDFT, and lower proportions of the sialylated HMOs, 3′SL and 6′SLN (Fig. 2B). The secretor-associated HMOs, 2′FL and LDFT, were strongly correlated between serum and urine. In 10 of 60 women, we detected only trace amount of 2′FL and LDFT in serum and urine samples, indicating secretor-negative status (Fig. 2C). Normalization of urinary HMOs with the osmolality resulted in a marked increase in *R*-squared values of the correlations between serum and urine for 2′FL, LDFT, and 3′SL (Fig. 2C).

We compared HMOs between cases and controls and found significantly higher LDFT concentrations in the serum and urine (Mann-Whitney *t* test) (Fig. 2D). Excluding the two women in the control group from the analysis who did not receive tocolytic treatment, but who delivered preterm, did not change results. Since short cervical length is associated with PTB, we then stratified all women regardless of experienced labor into two groups by cervical length (short versus normal) and found significantly higher serum levels of 3′SLN in women with a short versus normal cervix (Fig. 2D). Comparing women with subsequent PTB to women with term births in the total study group revealed significantly higher serum 3′SL levels with PTB, whereas urinary HMOs were not altered with PTB (Fig. 2D). Notably, also within the cases and thus controlling for the experienced labor and treatment with tocolytics, we found significantly higher serum 3′SL in women with PTB than with term births (see Fig. S1A in the supplemental material). In the case group, no other maternal characteristics were different between PTB and term birth, except for the prolactin concentration, which was lower in PTB compared to term birth (93.5 versus 139.9 ng/ml, *P* = 0.009).

10.1128/mSystems.00334-20.1 FIG S1 HMOs (A) and microbial taxa (B) were different in women with term births versus PTB when only case group was analyzed. (A) Similar to the whole study group, 3′SL was significantly higher in women with subsequent PTB compared to women who delivered at term. (B) LefSe analysis revealed L. crispatus RSVs correlated with PTB in vaginal microbiome, and L. crispatus and U. urealyticum RSVs correlated with PTB in urinary microbiome. Download FIG S1, PDF file, 0.3 MB. Copyright © 2020 Pausan et al. 2020 Pausan et al. This content is distributed under the terms of the Creative Commons Attribution 4.0 International license.

We next performed Spearman correlation analyses to explore associations between HMOs and potential indicators of PTB such as cervical length and inflammation markers, and other maternal factors, as well as with PTB outcome measured as GA at birth (Fig. 2E). We found that serum 2′FL and LDFT negatively correlated with vaginal pH (*r* = –0.26 [*P* = 0.032] and *r* = –0.22 [*P* = 0.009], respectively). Serum 3′SLN was negatively correlated with cervical length (*r* = –0.42, *P* = 0.001), and serum 3′SL was negatively correlated with GA at birth (*r* = –0.37, *P* = 0.004). All HMOs, especially serum LDFT and 2′FL, were positively correlated with GA at admission (Spearman rho *r* = 0.36 [*P* = 0.004] and *r* = 0.3 [*P* = 0.015], respectively). When we controlled for timing of sampling of the serum HMOs and adjusted the significance level using Bonferroni correction, the associations of LDFT with vaginal pH (*r* = –0.57, *P* < 0.0001) and of 3′SLN with cervical length (*r* = –0.55, *P* < 0.0001) remained significant (Fig. 2E). Serum 3′SL corrected for GA at sampling was still correlated with GA at birth (*r* = –0.37, *P* = 0.006) but did not pass the adjusted significance level.

### Microbial composition and density in vaginal and urinary samples is not indicative of preterm labor.

**FIG 3:**
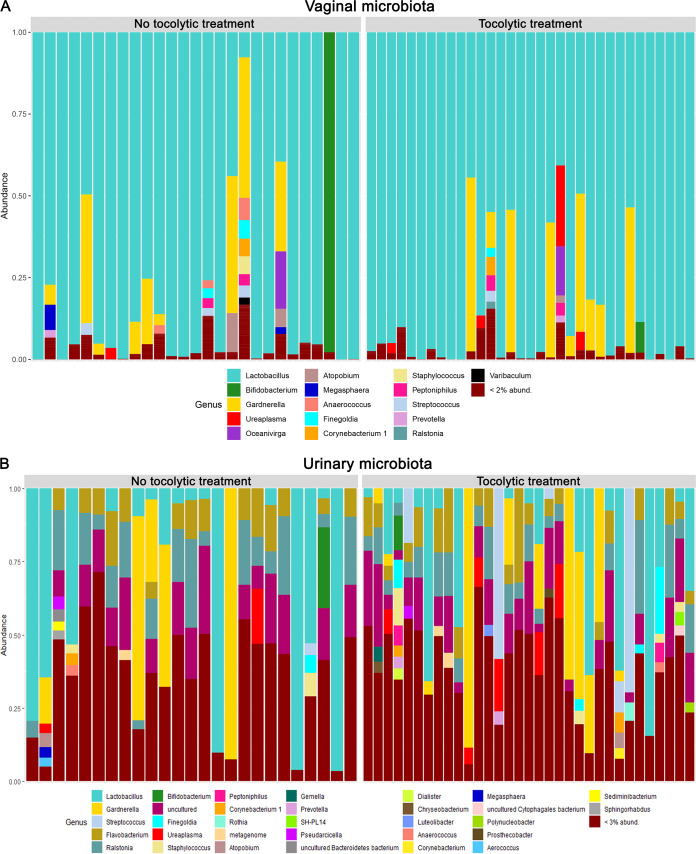
Microbial composition of urinary and vaginal microbiomes. (A) The vaginal microbial composition shows a dominance of *Lactobacillus* species. (B) The urinary microbiota is dominated by several different genera, such as *Lactobacillus*, *Gardnerella*, *Streptococcus*, and *Flavobacterium*.

We next used vaginal swabs and catheter urine for microbiome analysis. We found the vaginal microbiome to be dominated by *Lactobacillus*, followed by *Gardnerella*, *Atopobium*, *Ureaplasma*, *Anerococcus*, *Finegoldia*, and other genera (Fig. 3A), which is similar to results seen in previous studies (12, 30). The urinary microbiome was generally dominated by *Lactobacillus*, *Gardnerella*, *Streptococcus*, *Flavobacterium*, *Ralstonia*, *Prevotella*, *Ureaplasma*, and other microbial species, being similar to a healthy urinary microbiome (31) (Fig. 3B).

We next assessed differences in microbial diversity and density related to preterm labor, short cervix and PTB. Quantitative PCR (qPCR) results showed no significant differences in 16S rRNA gene copy numbers, either in the urine or the vagina, within the groups stratified into cases/controls, normal cervix/short cervix, or term birth/PTB (Fig. S2).

10.1128/mSystems.00334-20.2 FIG S2 Number of copies of microbial 16S rRNA gene of the vaginal and urine microbiota. (A, B) Number of 16S rRNA gene copies of vaginal (A) and urine (B) samples show no differences between the analyzed groups. Download FIG S2, PDF file, 0.09 MB. Copyright © 2020 Pausan et al. 2020 Pausan et al. This content is distributed under the terms of the Creative Commons Attribution 4.0 International license.

### Certain *Lactobacillus*, *Gardnerella*, and *Ureaplasma* signatures in the vaginal microbiome, but not necessarily community state types, correlate with preterm indicators and PTB.

**FIG 4:**
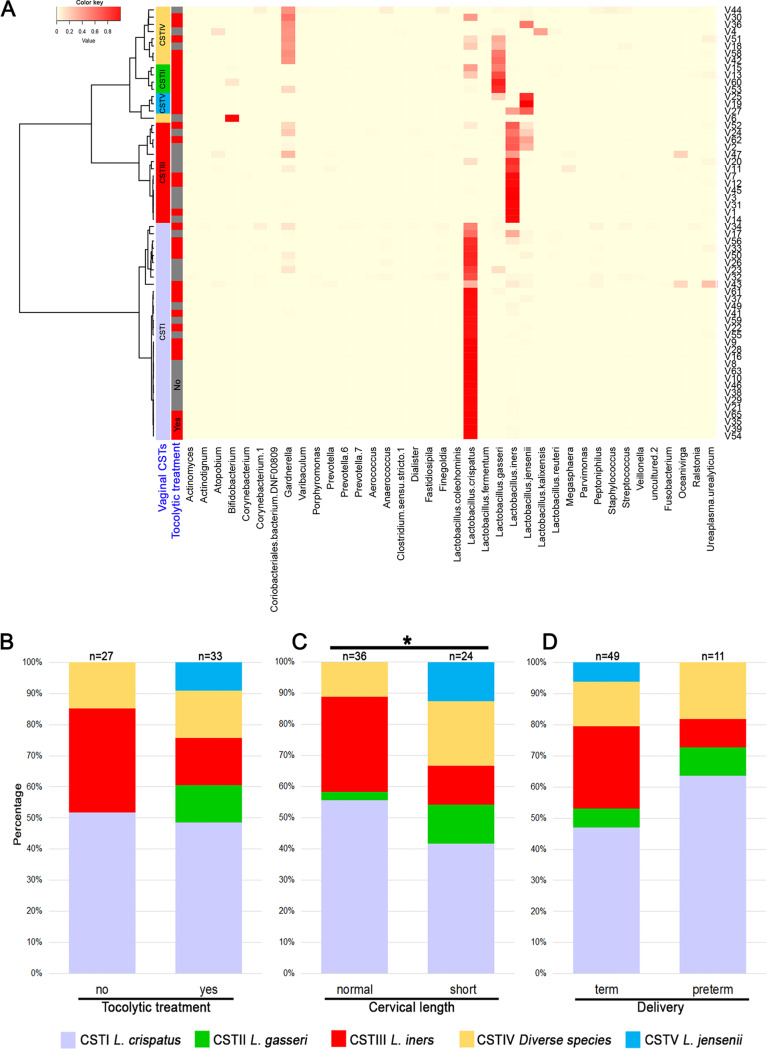
Vaginal microbiome clustered in community state types (CSTs) and the prevalence of CSTs in different groups. (A) Community state types as found in our cohort (heatmap). CSTs and their prevalence in the (B) tocolytic treatment group and (C) cervical length group show that CST VI is present only in women with preterm labor and those with a short cervix. (D) No specific vaginal CST was associated with delivery outcome.

Through hierarchical cluster analysis at species level, we classified vaginal samples into five CSTs (12) (Fig. 4A), characterized as follows: CST I (Lactobacillus crispatus), CST II (Lactobacillus gasseri), CST III (Lactobacillus iners), CST IV (diverse species), and CST V (Lactobacillus jensenii). The most prevalent CST was CST I (30/60, 50%), followed by CST III (14/60, 23%), CST IV (9/60, 15%), CST II (4/60, 7%), and CST V (3/60, 5%).

CST II and CST V were only present in cases, mainly in those with a short cervix, but not in subjects with PTB (Fig. 4B to D), indicating an association between L. jensenii and L. gasseri predominance, preterm labor, and short cervix. We found significant differences in the incidence of different CSTs in women with a normal versus a short cervix, but no differences between cases and controls or between term birth and PTB (Fig. 4B to D).

Analyzing the alpha and beta diversities of the vaginal microbiome in the three groups (tocolytic treatment, cervical length, and delivery outcome), we found no significant differences in diversity, richness, and clustering, indicating similar microbial communities within the groups (Fig. S3A to F).

10.1128/mSystems.00334-20.3 FIG S3 Alpha and beta diversities of the vaginal microbiome and a Spearman correlation heatmap at the genus level. (A to F) Alpha and beta diversities of the vaginal microbiome shows no differences between the tocolytic group (A and D), the cervical length group (B and E), and delivery outcome (C and F). (G) Spearman correlation heatmaps at the genus level of the vaginal microbiome show several associations between specific genera and cohort characteristics; for example, *Gardnerella* is associated with specific HMOs measured in the serum and urine and with the secretor status. Download FIG S3, PDF file, 9.8 MB. Copyright © 2020 Pausan et al. 2020 Pausan et al. This content is distributed under the terms of the Creative Commons Attribution 4.0 International license.

**FIG 5:**
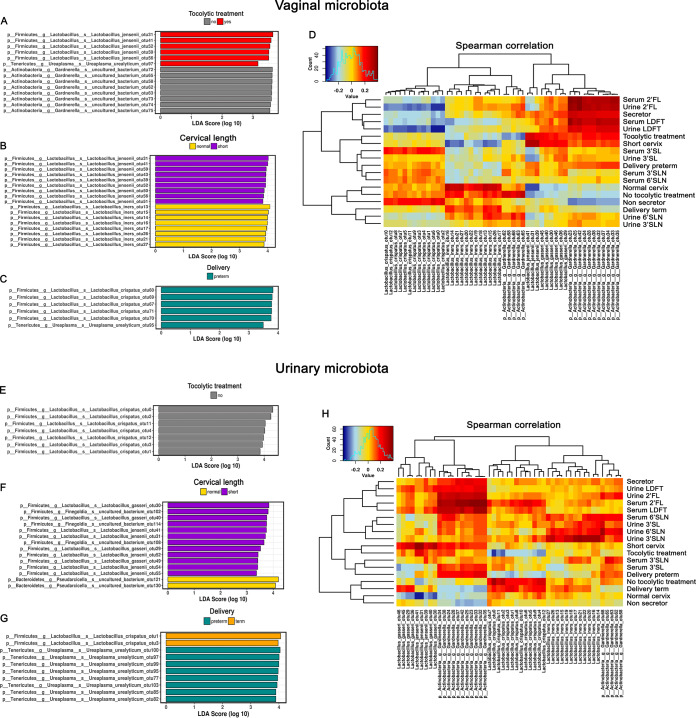
LEfSe analysis and Spearman correlation heatmaps of vaginal and urinary microbiome. (A to C) LEfSe (linear discriminant analysis effect size) values of vaginal microbiomes indicate associations of specific lactobacilli with preterm labor (A), cervical length (B), and delivery outcome (C). (D) A Spearman correlation heatmap of the same data (RSV level) shows associations between vaginal microbial signatures and different characteristics, such as HMOs, delivery outcome, and tocolytic treatment. (E to G) LEfSe values of urinary microbiomes indicate associations of specific lactobacilli with preterm labor (E), cervical length (F), and delivery outcome (G). (H) A Spearman correlation heatmap shows similar associations between specific *Lactobacillus* species and preterm labor, cervical length, and delivery outcome. Furthermore, *Gardnerella* species were associated with specific HMOs and PTB.

Next, we performed LEfSe (linear discriminant analysis effect size) to investigate associations between specific taxa and preterm contractions, cervical length, or PTB. Certain ribosomal sequence variants (RSVs) affiliated with L. jensenii were indicative for preterm contractions (RSV31, *P* = 0.038, analysis of variance [ANOVA]) and short cervix (*P* = 0.019) but not for PTB (*P* = 0.31), whereas L. iners was associated with normal cervix (RSV13, *P* = 0.041, ANOVA) and RSVs of L. crispatus with PTB. Ureaplasma urealyticum RSVs were correlated with PTB and preterm contractions (Fig. 5A to C). When we investigated the tocolysis group separately, we found, similarly to the total study group, that L. crispatus and U. urealyticum RSVs correlated with PTB (Fig. S1B).

In addition, we investigated associations between taxa (Fig. 5D) or genera (Fig. S2G) with cohort characteristics by Spearman correlation analysis and observed similar correlation patterns. RSVs of L. jensenii and L. gasseri strongly correlated with preterm labor and a short cervix. We found similar correlations between certain *Gardnerella* species and preterm labor, short cervix, and PTB, while other *Gardnerella* species were correlated with term delivery. At the genus level, certain genera, such as *Staphylococcus*, *Ureaplasma*, *Streptococcus* and *Finegoldia*, correlated with PTB. *Ureaplasma* was also correlated with tocolytic treatment but not with a short cervix (Fig. S3G).

### Microbiome signatures associated with vulnerability to PTB are also detectable in urinary samples.

In urine, we found no differences in the alpha (Fig. S3A to C) or beta (Fig. S4D to F) diversity of microbial communities within the three groups indicating similar microbial communities.

10.1128/mSystems.00334-20.4 FIG S4 Alpha and beta diversities of the urinary microbiome and a Spearman correlation heatmap at the genus level. (A to F) Alpha and beta diversities of the urinary microbiome shows no differences between the groups formed by tocolytic treatment (A and D), cervical length (B and E), and delivery outcome (C and F). (G) Spearman correlation heatmaps at genus level between the urinary microbiome and the cohort characteristics indicate specific correlations. For example, *Gardnerella* is associated with all measured HMOs and preterm delivery, and *Ureaplasma* is strongly correlated with preterm delivery. Download FIG S4, PDF file, 14.1 MB. Copyright © 2020 Pausan et al. 2020 Pausan et al. This content is distributed under the terms of the Creative Commons Attribution 4.0 International license.

LEfSe analysis identified several taxa to discriminate between the analyzed groups. RSVs of Lactobacillus jensenii (RSV41, *P* = 0.021, ANOVA), L. gasseri, and species of *Finegoldia* (RSV102, *P* = 0.043, ANOVA) were associated with a short cervix, whereas species of *Pseudarcicella* were associated with a normal cervix. L. crispatus was correlated with term deliveries and women who did not receive tocolytics (RSV0, *P* = 0.041, ANOVA), whereas U. urealyticum was associated with PTB (RSV100, *P* = 0.051, ANOVA) (Fig. 5E to G). We obtained similar LEfSe results when analyzing the tocolysis group only (Fig. S1B). Spearman correlation analyses between urinary microbiome and variables of the three groups revealed similar associations. In addition, specific species of *Gardnerella* were correlated with PTB, a short cervix, and preterm labor and all five HMOs investigated in serum and urine (Fig. 5H). At the genus level, *Gardnerella* was positively correlated with all measured HMOs, PTB, a short cervix, and tocolytic treatment. *Ureaplasma* was mainly associated with PTB and secretor-negative status, and *Pseudarcicella* was positively correlated with normal cervix (Fig. S4G).

### The urinary metabolites are unaffected by preterm labor.

Principal component analysis (PCA) plots of urinary metabolites revealed no differences between women with a short cervix or a normal cervix (Fig. S5) or between women with term birth or PTB (Fig. S6). Women treated with tocolytics had slightly decreased urinary citrate concentration compared to controls (Fig. S7C) but no differences in other metabolites (Fig. S7). We also compared the metabolites acetate, trimethylamine, and tyrosine, previously associated with PTB (32, 33), but we found no significant differences between cases and controls (data not shown).

10.1128/mSystems.00334-20.5 FIG S5 Metabolites of patient urine samples: short cervix versus normal cervix. PCA plots did not reveal any differences between patients with a short cervix compared to patients with normal cervix. Download FIG S5, PDF file, 1.7 MB. Copyright © 2020 Pausan et al. 2020 Pausan et al. This content is distributed under the terms of the Creative Commons Attribution 4.0 International license.

10.1128/mSystems.00334-20.6 FIG S6 Metabolites of patient urine samples: PTB versus term birth. PCA plots did not reveal any differences in patients with preterm births. Download FIG S6, PDF file, 1.7 MB. Copyright © 2020 Pausan et al. 2020 Pausan et al. This content is distributed under the terms of the Creative Commons Attribution 4.0 International license.

10.1128/mSystems.00334-20.7 FIG S7 Metabolites of patient urine samples: tocolytic treatment versus no treatment. When women receiving tocolytics are compared to untreated women, a slightly decreased citrate concentration was observed in the case group. Download FIG S7, PDF file, 1.7 MB. Copyright © 2020 Pausan et al. 2020 Pausan et al. This content is distributed under the terms of the Creative Commons Attribution 4.0 International license.

### The microbiome in urine and the vagina follows the HMO profile in serum and urine.

Having found differences in HMOs and certain microbial taxa related to preterm indicators and PTB, we sought to determine whether HMOs and the microbiome are linked. We identified strong correlations between vaginal and urine associated *Gardnerella* species and HMO concentrations in serum and urine. Specific vaginal genera such as *Ureaplasma*, *Gardnerella*, and *Flavobacterium* were positively correlated with concentrations of urine and serum LDFT and 2′FL and thus with secretor-positive status (Fig. S3G). Vaginal *Flavobacterium* genus was correlated with sialylated HMO concentrations in urine and with serum 3′SLN (Fig. S3G). Vaginal *Ureaplasma* was correlated with serum 6′SLN (Fig. S4G), whereas urine-associated *Ureaplasma* was correlated with sialylated HMO concentrations, with the exception of serum 3′SLN. Serum 3′SLN was mainly correlated with *Finegoldia* (Fig. S4G).

We also observed correlations between *Bifidobacterium* in urine and 6′SLN and 3′SL in urine and serum (Fig. S4G). Furthermore, we found correlations between specific HMOs and L. iners in urine (Fig. 5H). *Gardnerella* species in urine were also highly correlated with serum and urine LDFT and 2′FL concentrations and thus with positive secretor status. L. crispatus species were highly positively correlated with serum 3′SL concentration.

### Inflammation, either directly mediated by sialylated HMOs or indirectly through affecting the microbiome, might increase the risk for PTB.

Next, to investigate the microbial functions and their correlation with HMOs and metadata, we predicted functional profiles from our 16S rRNA gene data using Tax4Fun. Functional profiles of the urinary microbiome were categorized into three major groups (Fig. S8). Notably, one cluster of microbial functions was strongly influenced by secretor status, as reflected by an increase in sugar-degradation-related microbial functions (e.g., pentose and glucoronate interconversions [*P* = 0.031, ANOVA], pentosephosphate pathway, galactose metabolism, and starch and sucrose metabolism). This might explain the increase of particularly sugar-metabolizing microbial signatures, such as *Gardnerella*, along with secretor-associated HMOs LDFT and 2′FL.

10.1128/mSystems.00334-20.8 FIG S8 Estimated functional profiles of the urinary microbiome and correlations with HMOs and preterm delivery risk. Download FIG S8, PDF file, 1.2 MB. Copyright © 2020 Pausan et al. 2020 Pausan et al. This content is distributed under the terms of the Creative Commons Attribution 4.0 International license.

**FIG 6:**
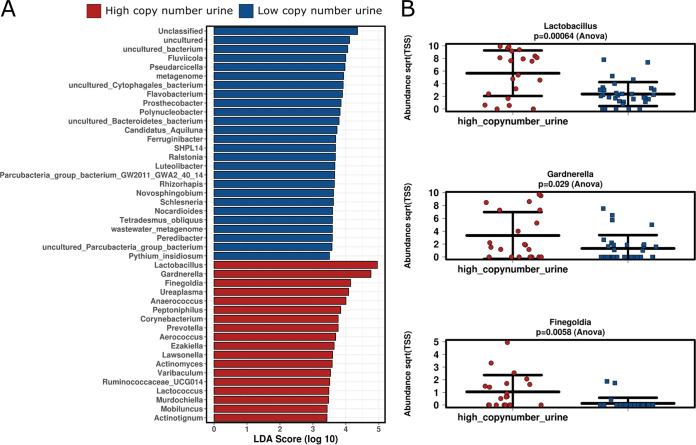
Distinct taxa associated with a high or low 16S rRNA gene copy number in the urinary microbiome. (A) LDA scores show significant bacterial differences at the genus level within the groups of high and low 16S rRNA gene copy numbers. (B) An increase in the abundance of *Gardnerella*, *Lactobacillus*, and *Finegoldia* was observed in the high-copy-number group.

Notably, microbiome functional profiles in the vagina and urine were not correlated with inflammation status (CRP), but microbial function profiles of urine, but not the vagina, were strongly correlated with the 16S rRNA gene copy numbers in urine samples (two categories of copy number groups, high and low, with a cutoff of 10^5^; *P* = 0.001). These copy numbers were increased along with—again—sugar-degrading microbial functions (galactose metabolism, starch and sucrose metabolism; LEfSe analysis) and corresponding microbial taxa, such as *Gardnerella* (*P* = 0.029), *Lactobacillus* (*P* = 0.00064), and *Finegoldia* (*P* = 0.0058, ANOVA) (Fig. 6), indicating an increased microbial load in urine due to secretor-associated HMOs. This was reflected also in the vaginal microbiome, since high copy numbers in urine correlated with an increase in *Gardnerella* and *Finegoldia* therein as well.

**FIG 7:**
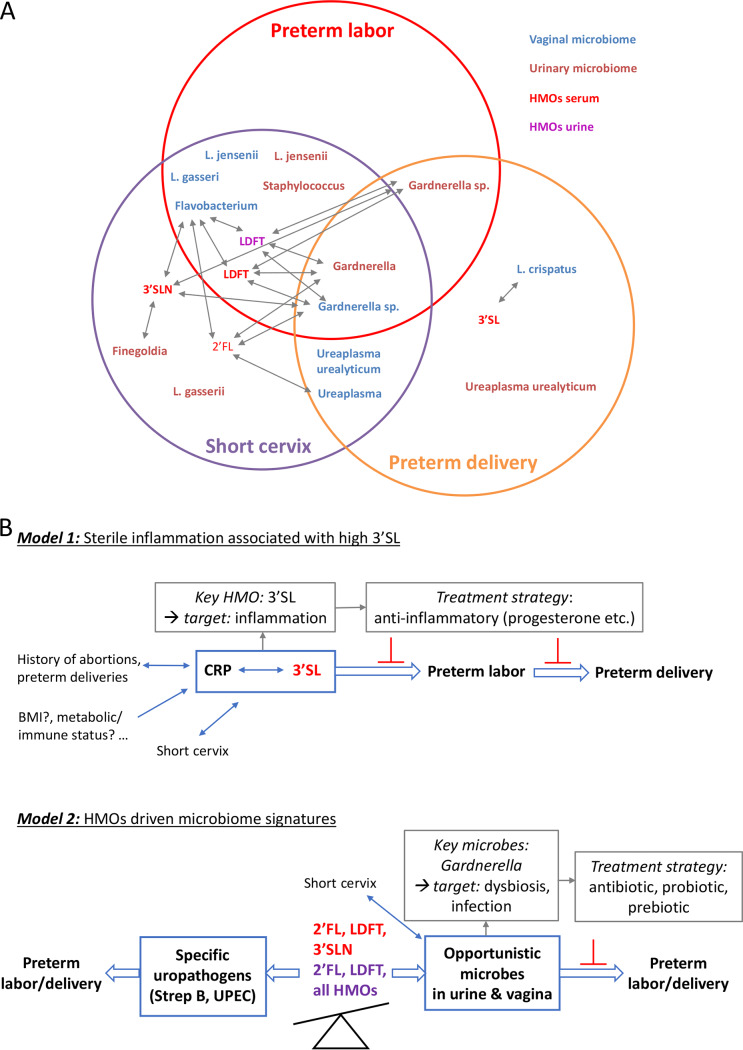
Overview of results and the proposed models. (A) A Venn diagram shows specific HMOs (red for serum and purple for urinary) and microbial genera or species (blue for vaginal and brown for urinary) that were found associated with preterm labor, a short cervix, or PTB and their interrelationships. Arrows indicate correlations between specific HMOs and microbes. (B) Two distinct models for how HMOs might be involved in modulating the risk for PTB and potential treatment strategies are shown. Model 1 is characterized by an increased concentration of sialylated HMOs, i.e., 3′SL, associated with sterile inflammation, independent of vaginal or urinary microbiome. Thus, anti-inflammatory therapies might be recommended. Model 2 is characterized by an imbalance in HMOs, leading to disturbances in the vaginal and urinary microbiomes. This, combined with other risk factors (a short cervix), might promote preterm labor and PTB. Although specific HMOs might prevent colonization with urogenital pathogens, the same HMOs might also foster the overgrowth of certain opportunistic microbial species in the urinary tract and then the vagina, leading to beginning infections and promoting preterm labor and PTB. 2′FL, 2′-fucosyllactose; LDFT, lactodifucotetraose; 3′SL, 3′-sialyllactose; 3′SLN, 3′-sialyllacosamine.

In Fig. 7, we present an overview of the results of our study, in which we identified potential key compounds and key microorganisms and their interplay, modulating the risk for PTB (Fig. 7A). Sialylated HMOs, in particular 3′SL, were positively correlated with PTB, a short cervix, and increased inflammation. Notably, 3′SL only partially correlated with microbial signatures in urine and the vagina, indicating an independent risk increase through a direct, but microbiome-independent mechanism (Fig. 7B). The key microbial signatures were L. jensenii, L. gasseri, *Ureaplasma* sp., *Gardnerella* sp., and *Finegoldia* sp., which appeared to correlate with a short cervix, PTB, and/or preterm labor. Notably, most of these microorganisms were associated with an increased bacterial load in urine, indicating potential low-grade inflammation or a beginning infection therein. Particularly, *Gardnerella* signatures were associated with secretor status, in agreement with observations made on a functional level. A positive secretor status was also found to be associated with a higher bacterial load in urine.

To summarize, our observations point to two different processes. One process appears to be driven by sterile inflammation, characterized by increased sialylated HMO concentrations in serum, and another process appears to be indirect and microbiome mediated, which could, however, be driven by secretor-associated HMOs (Fig. 7B).

## DISCUSSION

Our study combines comparative analyses of serum and urinary HMOs, vaginal and urinary microbiomes, and urinary metabolites in pregnancies at risk for spontaneous PTB and in healthy controls. We identified HMOs and microbial signatures associated with a higher risk of PTB, pointing toward novel roles of HMOs in regulating a healthy, term pregnancy.

In this exploratory study, we found that elevated serum 3′SL (independent of labor and GA) is associated with PTB. While some studies have investigated changes in HMOs in human milk as a consequence of PTB (34–36), their potential causal role in PTB has not yet been explored. Recently, we showed that serum HMOs change during pregnancy (15, 16) and that an individual HMO, 3′SL, may be predictive of gestational diabetes mellitus (GDM) (16). An early study on maternal urinary HMOs speculated about the importance of a balanced HMO profile in maintaining a healthy pregnancy (17). Our results seem to support this hypothesis, as higher 3′SL was associated with risk for PTB. We also found an association of 3′SL with PTB when analyzing the cases only, ruling out experienced labor and respective treatment as confounders.

In this cross-sectional study, the sampling window was from 23^+0^ to 34^+0^ weeks of gestation, and especially the serum concentration of secretor-associated HMOs were significantly positively correlated with GA. Correcting for GA at sampling, we found a strong negative association of serum 3′SLN with cervix length and of 3′SL with PTB. Interestingly, 3′SLN and 3′SL in serum, but not in urine, were associated with a short cervix or PTB. Discrepancies in these associations with HMOs in serum versus in urine could be due to multiple factors, e.g., attributed to the relatively small sample size, or to errors in the normalization of urine. Sialylated HMOs were also less correlated between serum and urine, potentially because of specific transport into urine or (microbial) degradation in urine. All of this might indicate a more systemic, microbiome-independent mechanism by which sialylated HMOs modulate the risk of PTB.

Our finding that sialylated HMOs in serum are associated with a short cervix and PTB is in accordance with a study reporting an increased total serum sialic acid concentration associated with PTB (37). In human milk, HMOs were also found to be different in sialylation after PTB (35, 36). These findings support the hypothesis that sialyation, in general, is altered in preterm labor mediated by (sterile) inflammatory processes. This pathway would not necessarily be dependent on changes in the urinary and vaginal microbiomes and would explain why urinary HMOs are not associated with PTB in our cohort excluding clinically manifest infections. The positive associations of sialylated HMOs with leukocytes and CRP support an inflammatory component in PTB. The inflammation-associated increase in serum 3′SL seems to fit with our previous study in pregnant women at risk for GDM, which is also considered an inflammatory disorder. Serum 3′SL was higher in women who later developed GDM (16). In addition, accumulating evidence points toward increased plasma sialic acid in inflammatory pathologies such as cancer, cardiovascular diseases, and type 2 diabetes (38). Inflammation was associated with altered glycosylation due to extrinsic sialylation via extracellular sialyltransferases (39). Whether increased sialylation is a general response to inflammatory conditions in pregnancy and whether changes in sialylated HMOs have ameliorating or deteriorating effects on the pregnancy remain to be elucidated. Moreover, we need accordingly designed larger prospective studies to investigate the potential of sialylated HMOs in serum early in pregnancy as predictive markers for PTB.

Another main finding of this study are the associations of HMOs with specific genera and species of the urinary and vaginal microbiomes. Our results indicate that HMOs in urine (and serum) shape microbial communities, supporting in particular microbes capable of metabolizing HMOs. Like bifidobacteria, Gardnerella vaginalis possess the fructose-6-phosphate phosphoketolase (F6PPK), the key enzyme of the so-called “bifid shunt,” a specific pathway for carbohydrate metabolism (40, 41). In accordance with our estimation on functional capacities, this indicates that *Gardnerella* can utilize HMOs, in particular those associated with secretor-positive status. This leads us to speculate about a potential adaptation and growth advantage of *Gardnerella* in secretor-positive, pregnant women, opening up new questions on longitudinal associations with gestation and increasing HMO concentrations.

Previous studies have reported a decrease in vaginal microbial diversity and richness between the first and second trimesters in women with PTB (42), while other studies could not identify changes in microbial diversity and richness (10, 30, 43). In our study, we did not observe significant differences in diversity, richness, or bacterial load in women at increased risk. However, since we sampled at a GA when PTB can occur, we might have missed potential preceding microbial changes earlier in pregnancy.

We identified CST V, dominated by Lactobacillus jensenii, mainly in cases and especially in women who had a short cervix. Interestingly, none of the women with PTB had CST V at the time of sampling. In addition, we observed associations between Ureaplasma urealyticum or species of *Gardnerella* with preterm labor and short cervix. Others have shown associations between either specific taxa (*L. iners*) or vaginal CSTs (CST IV) and PTB (8, 10, 11, 43).

Our findings that L. jensenii and *Gardnerella* abundance in the vaginal microbiome was associated with higher risk for PTB are in accordance with previous studies (43, 44). However, we could not confirm previous observations that vaginal microbiome dominated by L. crispatus is protective against PTB (10, 43, 44). In contrast, 7 of 11 women who delivered preterm had a vaginal microbiome dominated by L. crispatus. We are aware that the number of women with PTB in our cohort is relatively small compared to other studies, and larger cohort studies are needed to validate these results.

Our results on the urinary microbiome are consistent with a previous study, finding no changes in the alpha and beta diversities of the urinary microbiome between women delivering preterm and at term (14). However, we observed enriched taxa between the groups, e.g., a higher relative abundance of L. crispatus in controls and in women with term births. L. gasseri, L. jensenii, and species of *Finegoldia* were enriched in women with short cervix, whereas U. urealyticum was increased in women with PTB. We also observed strong correlations between PTB or a short cervix and species of *Gardnerella*. Since none of the women showed symptoms of UTIs at the time of sampling, the increase in U. urealyticum in the PTB group speaks for asymptomatic UTIs which could be monitored as a risk factor. In contrast to our study, Ollberding et al. have shown an increase in the relative abundances of *Prevotella*, *Sutterella*, *L. iners*, *Blautia*, *Kocuria*, *Lachnospiraceae*, and S. marcescens, which might be due to differences in ethnicities (14).

Previous studies found hormones associated with changes in microbiome potentially influencing the risk for PTB (45, 46). Here, the hormones estrogen, progesterone, and prolactin were not different in cases versus controls or in women experiencing PTB versus a term birth (data not shown) within the total study group. None of the investigated hormones was associated with specific microbial species or genus, speaking against a significant influence on the microbiome in our study population.

Our study aimed to investigate medically unexplained preterm labor. Thus, we excluded women diagnosed with, or previously treated for UTI, which is causally linked to preterm labor and PTB. Previous *in vitro* studies have shown that HMOs protect against uropathogenic Escherichia coli invasion and cytotoxicity (47) or induce growth defects in group B streptococcus (48), both common uropathogens associated with UTI. Secretor-negative status was associated with higher incidence of UTI in pregnant women (49), indicating that secretor-associated structures at urothelial sites might protect from UTI. This seems to partially contradict our finding that secretor-positive status was associated with increased bacterial load in urine, correlating with an increase in *Gardnerella* and *Ureaplasma* signatures. However, having excluded symptomatic UTI might have biased our collective, leading to a smaller proportion of secretor-negative women at risk for PTB than normally found in the general population.

### Strengths and limitations.

A particular strength of our study is the innovative and comprehensive design, including different approaches (e.g., measurement of HMOs in urine and blood, NGS for vaginal and urinary microbiome, and measurement of urinary metabolites) to understand possible differences in pregnancy in women at high risk for preterm labor. Another strength is the consideration of host factors, pregnancy hormones, and the exclusion of previous antibiotic treatment and probiotics. Although previous studies have focused on mostly vaginal microbiome or, to a lesser extent, on the urinary microbiome, we included here both microbial communities. Importantly, we investigated here for the first time novel pregnancy factors, HMOs, present in serum and urine. Introducing HMOs in pregnancy will stimulate future studies investigating how HMOs can shape the urinary and vaginal microbiome. By including association analyses of specific hormones, inflammatory markers, and all relevant clinical data, we were able to estimate influence of these potential modulators of risk.

However, we are aware of some limitations of the study. The data presented here are mostly correlational and thus do not allow conclusions about causality or the nature of mediators of these associations. Our study was designed as a cross-sectional pilot study, comparing women with or without labor at a defined GA; however, over a relatively long window of time (weeks 23 to 34). While this design allowed comparing groups of women with higher risk (based on confirmed preterm labor or cervical length) and investigating bivariate correlations while controlling for GA, the sample size was not powered to investigate the outcome PTB versus term birth. Controlling for multiple confounders (secretor status, history of preterm births, miscarriages, BMI, smoking, and hormonal status) using multivariate regression models was not applicable due to the small sample size. However, despite the relatively low total sample size and due to the sampling in a higher-risk population presenting with potential preterm labor, the percentage of PTB was higher than in the general population. This offered interesting insights into an inflammatory etiology of PTB in this population, which is less treatable by anti-infective measures. In this context, it would be interesting to monitor effects of the tocolysis and anti-infective treatment on HMOs and the microbiome. Moreover, future studies should also use longitudinal prospective pregnancy studies covering blood and microbiome sampling also in the first and second trimester.

### Conclusions.

In conclusion, our results show that the concentrations of specific HMOs and several microbial signatures in both vaginal and urinary microbiomes are associated with preterm labor, a short cervix, and PTB. We observed strong correlations between sialylated HMOs, especially 3′SL, and a short cervix, PTB, and inflammation. In the vaginal microbiome, we identified *Gardnerella* sp. as being associated with preterm labor, a short cervix, and PTB. *Ureaplasma* sp. correlated with a short cervix and PTB, and L. jensenii, L. gasseri, and *Flavobacterium* were associated with preterm labor, whereas L. crispatus was correlated with PTB. In the urinary microbiome, we detected changes in similar taxa. *Gardnerella* was the only taxon associated with preterm labor, a short cervix, and PTB, whereas other microbial signatures were associated only with PTB (e.g., Ureaplasma urealyticum), only with a short cervix (*Finegoldia* and L. gasseri), or with both preterm labor and a short cervix (L. jensenii and *Staphylococcus*).

Overall, our observations highlight two different mechanisms involved in the etiology of preterm labor and PTB. One seems to be driven by sterile inflammation, characterized by increased sialylated HMO concentrations in serum, and the second mechanism appears to be indirect and microbiome mediated, which could, however, be driven by secretor-associated HMOs.

Our results identifying HMOs and key microbial taxa associated with PTB will guide current efforts to better predict the risk for PTB in seemingly healthy pregnant women and also provide appropriate preventive strategies.

## MATERIALS AND METHODS

### Experimental design and clinical metadata.

A total of 60 pregnant women were recruited with suspected preterm labor. Study subjects had a viable pregnancy of greater than 23^+0^ weeks of gestation, but not more than 34^+0^ weeks of gestation, were healthy, 18 years of age or older, and willing to consent to all aspects of the protocol. Subjects were excluded if they had any recent genitourinary infections, multiple pregnancy, or more than three consecutive miscarriages, any known fetal anomalies associated with possible growth or genetic anomalies, prepregnancy diabetes type 1 or 2 or gestational diabetes mellitus, prepregnancy hypertension, or if any antibiotic/prebiotic treatment was administered in the last 6 months. Clinical metadata are listed in Table S1 in the supplemental material. Gestational age is provided as weeks and days. To diagnose preterm labor, subjects underwent (i) cardiotocography (CTG) to determine the uterine contractions and their frequency and (ii) transvaginal ultrasonography for the measurement of the cervical length. Tocolysis was administered when ≥4 contractions/30 min clearly felt by the patient were recorded by CTG. Based on the CTG examination and subsequent decision to give tocolysis, we grouped the women into cases (pregnant women who received tocolytics) or controls (pregnant women who did not receive tocolytics). As tocolytic treatment, all women in the case group received Tractocile 6.75 mg/0.9 ml solution for injection (Atosiban) in the recommended dosage as bolus injection, followed by infusion. Two women additionally received Gynipral (Hexoprenalin) intravenously. Tocolysis treatment was not given when medically contraindicated, e.g., when the gestational age was >33 completed weeks or with premature rupture of membranes at >30 weeks of gestation. Two women in the control group did not receive tocolysis because of these contraindications.

### Sample collection and processing.

Blood, urine, and vaginal specimens were collected by trained personnel after recruitment and before any medical treatment. Urine specimens were collected using urinary catheters in sterile urine tubes. Vaginal swabs were collected using FLOQSwabs (Copan, Milan, Italy). All specimens were stored at 4°C, divided into aliquots within 4 h after collection, and stored at –80°C until further processing.

Genomic DNA was extracted from urine and vaginal specimens using a QIAamp DNA minikit (Qiagen) with some modifications. Before extraction with the kit, urine samples were centrifuged at 4,400 × *g* for 15 min; the supernatant was removed except for 500 μl that was used to resuspend the pellet. Then, 500 μl of lysis buffer (sterile filtered, 20 mM Tris-HCl [pH 8], 2 mM sodium EDTA, 1,2% Triton X-100) was added to the vaginal swabs. To both the vaginal and urine samples, 50 μl of lysozyme (10 mg/ml) and 6 μl of mutanolysin (25 KU/ml) was added, followed by incubation at 37°C for 1 h. The obtained mix was transferred to lysing matrix E tubes (MP Biomedicals), followed by a mechanical-lysis step at 5,500 rpm for 30 s two times using a MagNA Lyser instrument (Roche, Mannheim, Germany). After mechanical lysis, the samples were centrifuged to separate the beads from the supernatant at 10,000 × *g* for 2 min. Afterward, the DNA was extracted according to the provided instructions. The DNA was eluted in 100 μl of elution buffer for vaginal samples and in 60 μl for urine samples. Genomic DNA concentration was measured by using Qubit HS. Most urine samples had DNA concentrations below the detection limit.

### PCR and qPCR amplification.

Genomic DNA was used to amplify the V4 region of the 16S rRNA gene using the Illumina-tagged primers 515FB and 806RB (50). The PCR was performed in a final volume of 25 μl containing the following: TaKaRa Ex Taq buffer with MgCl_2_ (10×; TaKaRa Bio, Inc., Tokyo, Japan), primers at 200 nM, a deoxynucleoside triphosphate mix at 200 μM, TaKaRa Ex Taq polymerase (0.5 U), water (Lichrosolv; Merck, Darmstadt, Germany), and DNA template. For vaginal samples, 20- to 25-ng portions were used, while for urine 3 to 4 μl of genomic DNA was used. The PCR amplification conditions were as follows: initial denaturation for 3 min at 94°C, denaturation for 45 s at 94°C, annealing for 1 min at 50°C, elongation for 1 min 30 s at 72°C (38 cycles), and final elongation for 10 min at 72°C.

The numbers of bacterial 16S rRNA gene copies were determined using a SYBR-based approach with the primers Bac331F and Bac797R (51). For qPCR, the reaction mix contained 1× SsoAdvanced Universal SYBR Green Supermix (Bio-Rad, Hercules, CA), 300 nM concentrations of forward and reverse primer, gDNA template (20 to 25 ng for vaginal samples, 1 μl of gDNA for urine samples), and water (Lichrosolv; Merck). The qPCR was performed using a CFX96 Touch real-time PCR detection system (Bio-Rad). The qPCR conditions were as follows: initial denaturation for 15 min at 95°C, denaturation for 15 s at 94°C, annealing for 30 s at 54°C, elongation for 40 s at 72°C (40 cycles), and a melting curve at 60 to 95°C. All qPCRs were performed in triplicates. Crossing point (Cp) values were determined using the regression method within the Bio-Rad CFX Manager software, version 3.1. Absolute copy numbers of bacterial 16S rRNA genes were calculated using the Cp values and the reaction efficiencies based on standard curves obtained from defined DNA samples from E. coli (52). The qPCR efficiency was between 90 and 105%, and the *r*^2^ values were always >0.9. The detection limits were defined based on the average Cp values of nontemplate controls (triplicates) and the corresponding standard curves of the positive controls.

The bacterial 16S rRNA gene copies were determined using a SYBR-based qPCR (primer pair, Bac331F-Bac797R).

### Amplicon sequencing, bioinformatics, and statistical analysis.

Library preparation and sequencing of amplicons were carried out at the Core Facility Molecular Biology at the Center for Medical Research at the Medical University of Graz, Austria, as previously described (53). Obtained MiSeq data are available in the European Nucleotide Archive under the study accession number PRJEB31915.

MiSeq data analysis was performed using QIIME2 (54) as previously described (55). Taxonomy was assigned using the SILVA v132 database (56). The obtained feature table and taxonomy file were used for further analysis (see Table S1 in the supplemental material). The sequences classified as *Lactobacillus* genus were used to classify to the species level, allowing the clustering of the vaginal microbiome into CSTs based on hierarchical clustering. The classification of the *Lactobacillus* genus to species level was performed by using EzBioCloud (57). To further test that the classification is correct, a maximum-likelihood tree was constructed in MEGA7 (58) using the sequences classified into the *Lactobacillus* genus. The alignment was performed in SINA (59), and the closest neighbors were picked only from the All-Species Living Tree project (60).

Hierarchical clustering, alpha-diversity indices, principal coordinate analysis (PCoA) plots, and bar plots were assessed in R on the relative abundance at the species level based on Bray-Curtis dissimilarity and the method ward using the packages vegan (61) and gplots (62). Alpha-diversity indices were calculated using vegan package in R. Plots of the alpha-diversity indices were generated in R using the ggplot2 package (63). Differences in the alpha-diversity indices between the groups were tested in R using the Wilcoxon rank test. PCoA plots were constructed based on Bray-Curtis dissimilarities using phyloseq (64) and ggplot2 packages in R. The differences in the microbial composition between the analyzed groups were tested in R using the Adonis test. Bar plots based on the relative abundance at the genus level were developed for both the urinary and the vaginal microbiomes using ggplot2. LEfSe analysis plots and correlation heatmaps were generated using Calypso (65). Microbial functional profiles from 16S rRNA gene data were predicted by Tax4fun (66). The results were visualized using Calypso. All statistical analyses were performed in SPSS (IBM Corp.) unless stated otherwise.

### Metabolomics.

Urinary metabolites were analyzed using NMR, performed at 310K on an Avance Neo Bruker Ultrashield 600 MHz spectrometer equipped with a TXI probe head. The phosphate buffer solution used for nuclear magnetic resonance (NMR) analyses was prepared by dissolving 102 g of anhydrous KH2PO4 (VWR, Darmstadt, Germany), 0.5 g of TSP [3(trimethylsilyl) propionic acid-2,2,3,3-d4 sodium salt; Alfa Aesar, Karlsruhe, Germany], and 0.065 g of NaN3 (VWR) in 500 ml of D_2_O (Cambridge Isotope Laboratories [Tewksbury, MA] and adjusted to pH 7.4 with 1 M NaOH [VWR] and HCl [VWR]). Then, 300-μl portions of this NMR buffer in D_2_O were added to samples containing 200 μl of urine and transferred to 5-mm NMR tubes. A 1D CPMG (Carr-Purcell-Meiboom-Gill) pulse sequence (cpmgpr1d; 128 scans; 73,728 points in F1; 11,904.76-Hz spectral width; 128 transients; recycle delays, 4 s) with water suppression using presaturation was used for ^1^H 1D NMR experiments. Bruker Topspin version 4.0.2 was used for NMR data acquisition. The spectra for all samples were automatically processed (exponential line broadening of 0.3 Hz), phased, and referenced using TSP at 0.0 ppm and Bruker Topspin 3.5 software (Bruker GmbH, Rheinstetten, Germany).

Integral regions for metabolites of interest corresponding to a certain number of protons and for an external standard were defined. Concentrations of compounds were determined using Chenomx NMR Suite 8.3 with an internal standard concentration. All quantified metabolites were normalized to the original concentration of the urine.

For multivariate statistical analysis, NMR data were imported into Matlab vR2014a (MathWorks, Natick, MA), regions around the water and TSP signals were excluded, and probabilistic quotient normalization was performed to correct for differences in sample metabolite dilution. MetaboAnalyst (4.0) was the performed to identify changes in metabolites in the urine samples.

### HMO isolation and analysis.

Oligosaccharides from maternal serum were isolated as previously described (15) using chloroform-methanol extraction, solid-phase extraction with C_18_ columns, and graphitized carbon columns. Urinary HMOs were isolated by subjecting 10-μl urine samples and an internal standard directly onto C_18_ columns and then following the protocol used for the serum samples.

Isolated HMOs were fluorescently labeled (2-aminobenzamide) and separated by HPLC with fluorescence detection as described previously (26). Commercially available standards for 2′-fucosyllactose (2′FL), lactodifucotetraose (LDFT), 3′-sialyllactose (3′SL), 3′-sialyllactosamine (3′SLN), and 6′-sialyllactosamine (6′SLN) (Prozyme, Hayward, CA) were used to annotate HPLC peaks.

To normalize the HMO concentration in urine samples, the total osmolality indicative of the solute concentration was measured in urine samples by using a cryoscopic osmometer (Osmomat 030; Gonotec, Berlin, Germany). The HPLC chromatogram areas under the curve for individual HMO peaks were divided by osmolality and multiplied by the mean osmolalities for all urine samples.

Secretor status was determined based on 2′FL and LDFT abundance. HMO data are presented as the median and interquartile range for skewed data. Changes in HMOs between groups were tested by using s Mann-Whitney U test, and graphs were plotted using Prism (v8.00; GraphPad Software, La Jolla, CA).

### Data availability.

The obtained MiSeq data are available in the European Nucleotide Archive under the study accession number PRJEB31915.
