# Supplementary figures and images for: Human Milk Oligosaccharides Modulate the Risk for Preterm Birth in a Microbiome-Dependent and -Independent Manner
Source: mSystems. 2020 Jun 9;5(3):e00334-20. doi: 10.1128/mSystems.00334-20 (PMC7289590; doi:10.1128/mSystems.00334-20)

A

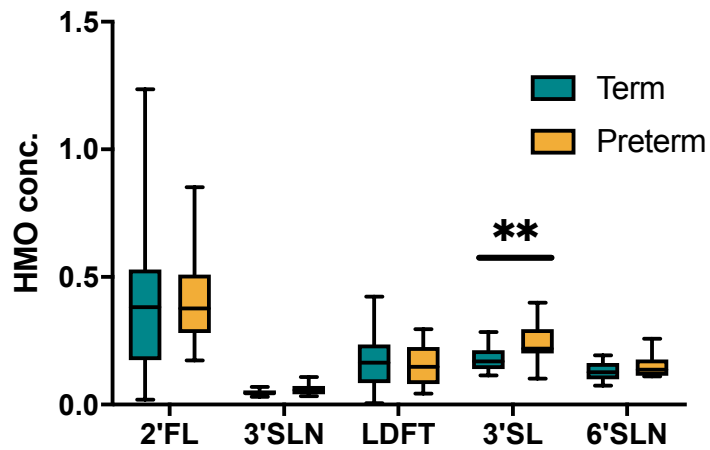

B

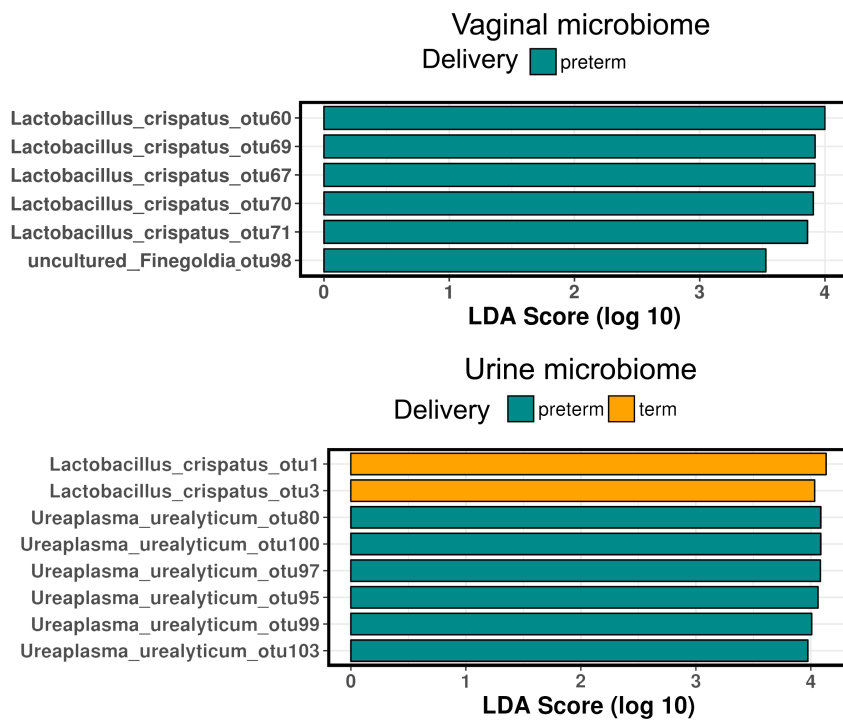

Supplement: FIG S1 [file mSystems.00334-20-sf001.pdf]

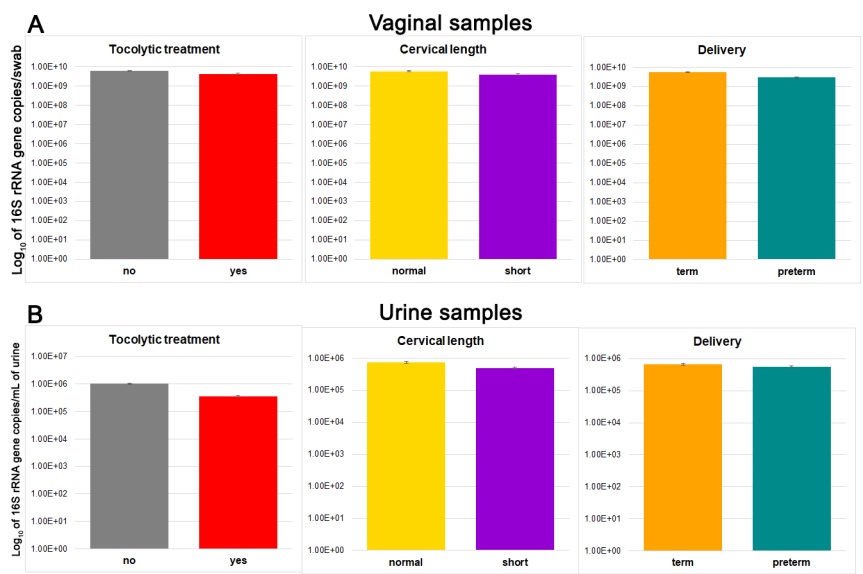

Supplement: FIG S2 [file mSystems.00334-20-sf002.pdf]

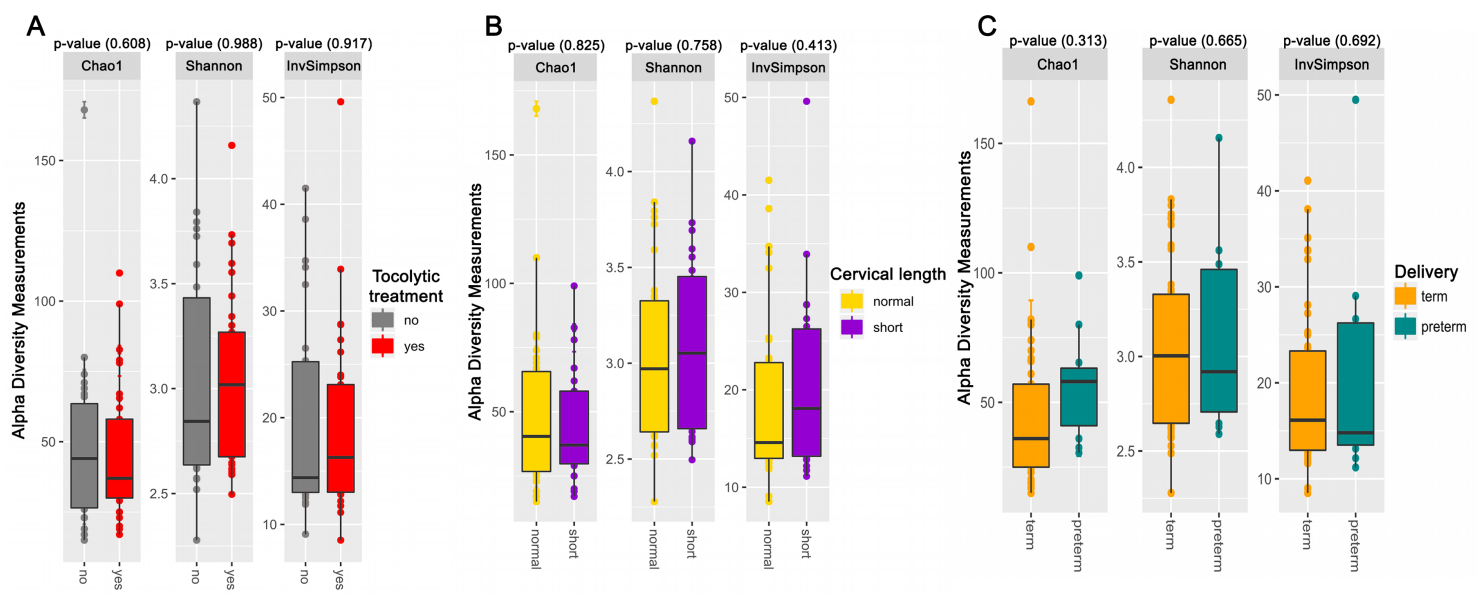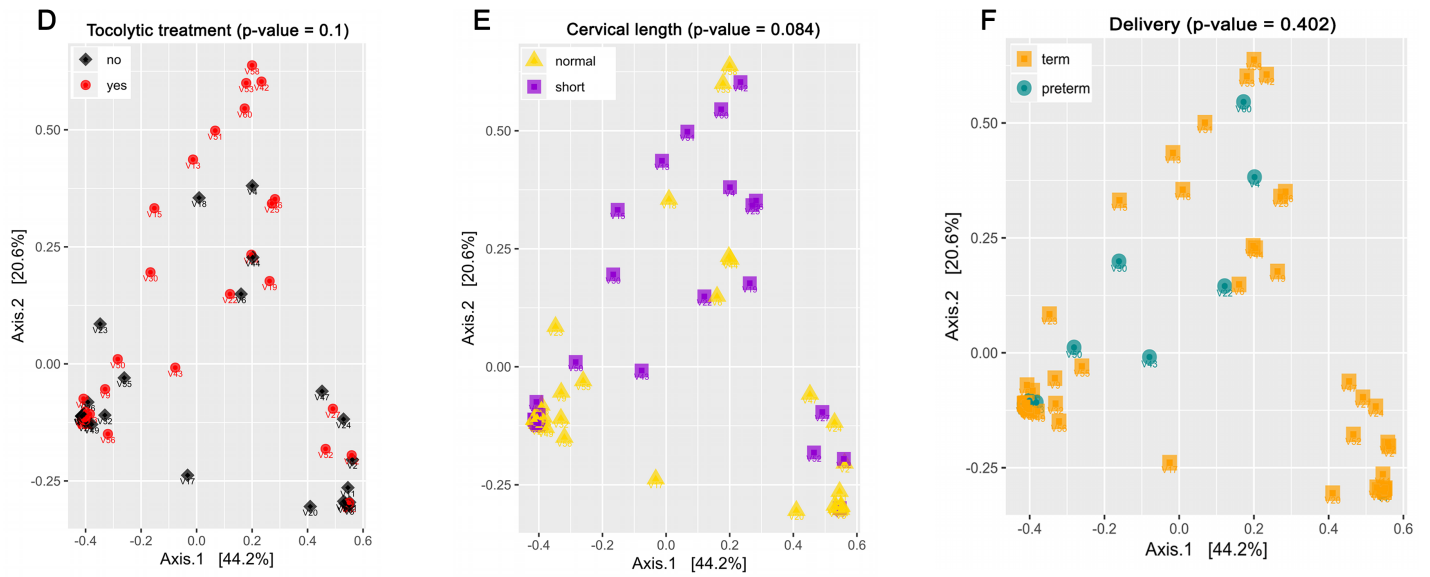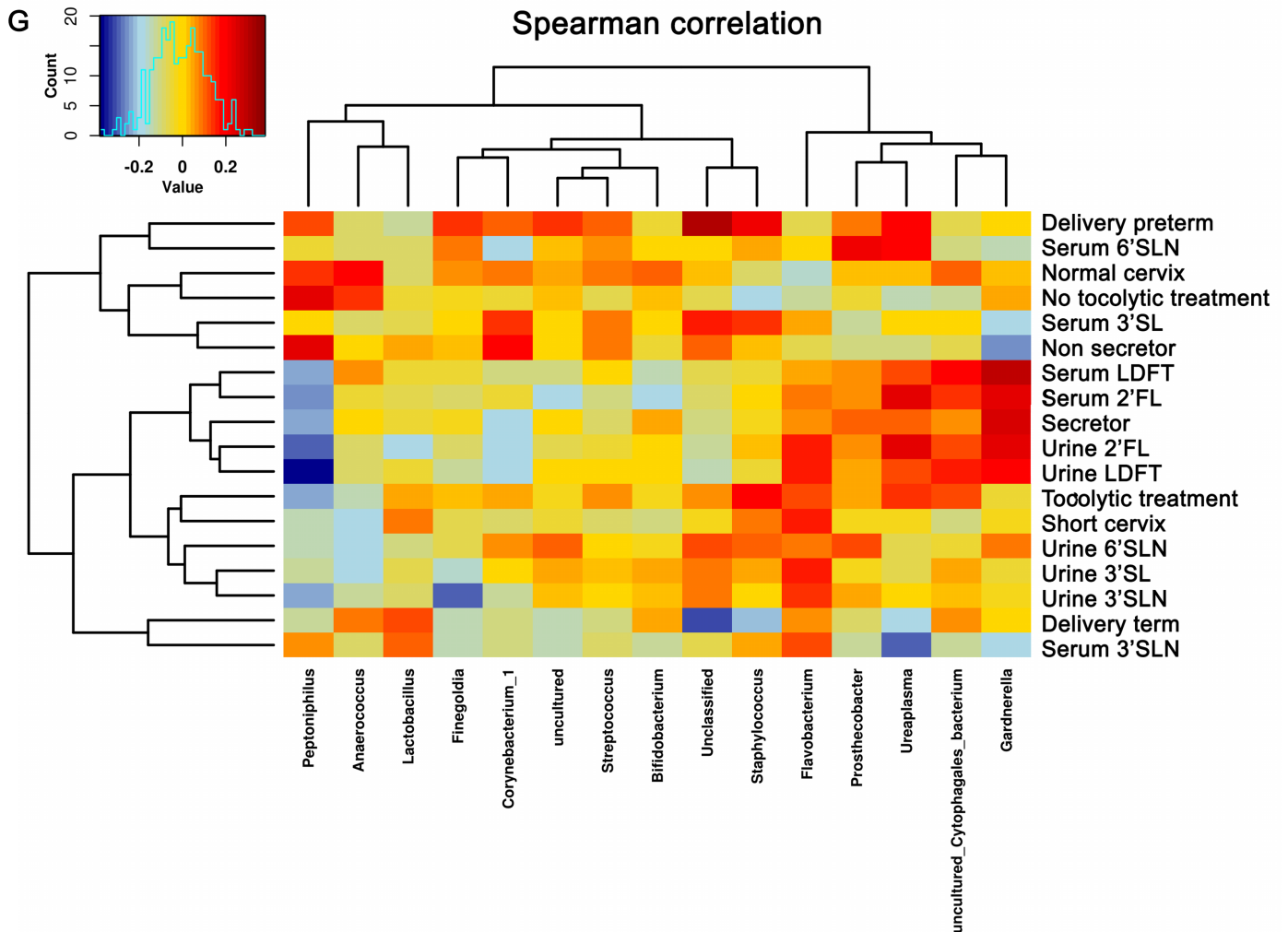

Supplement: FIG S3 [file mSystems.00334-20-sf003.pdf]

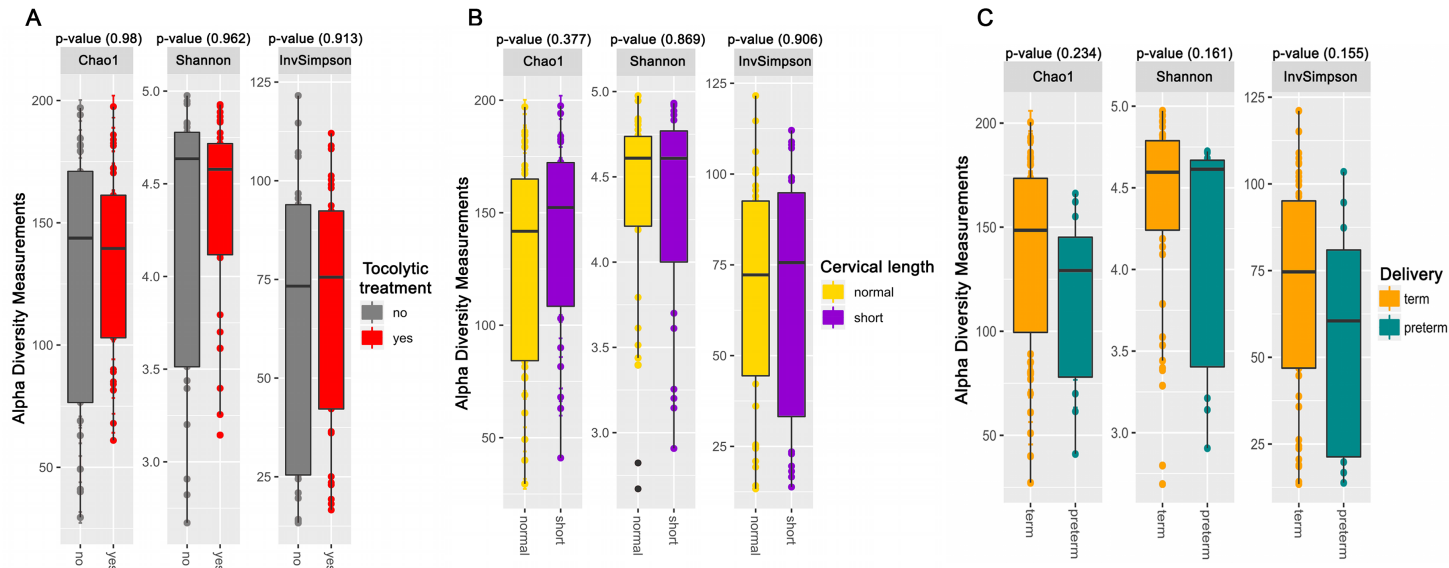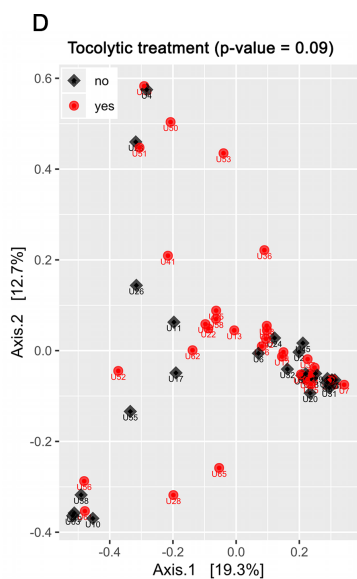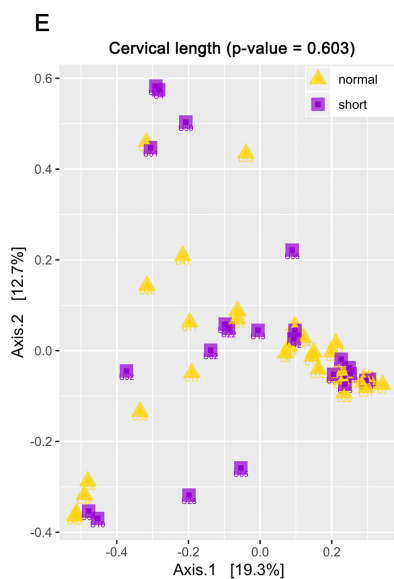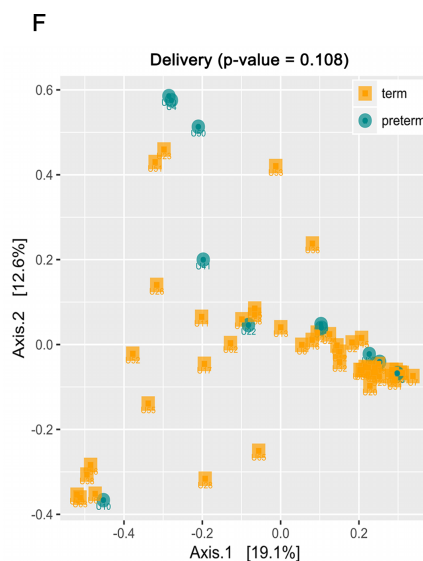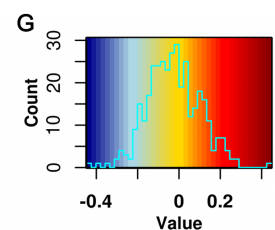

## Spearman correlation

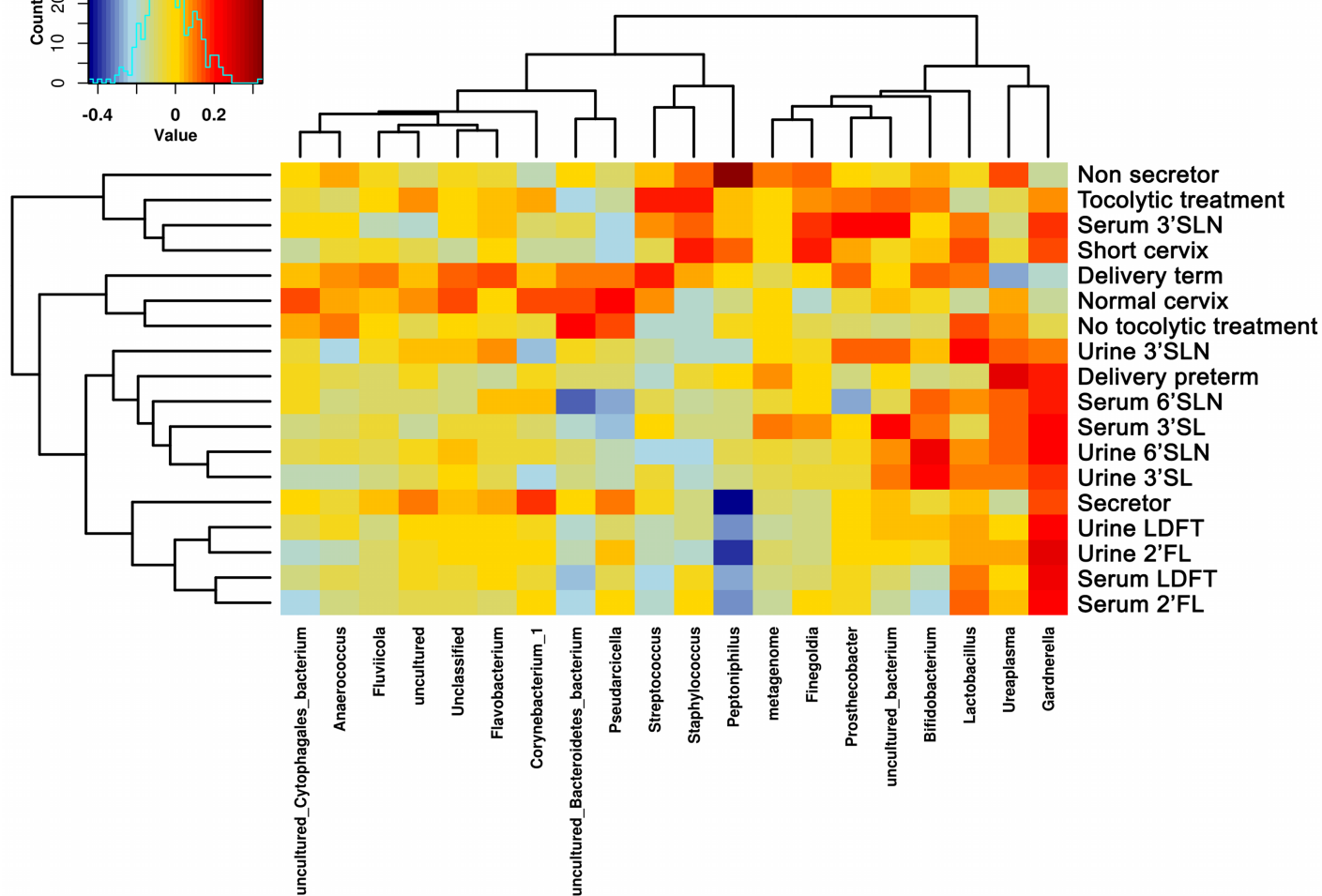

Supplement: FIG S4 [file mSystems.00334-20-sf004.pdf]

**A**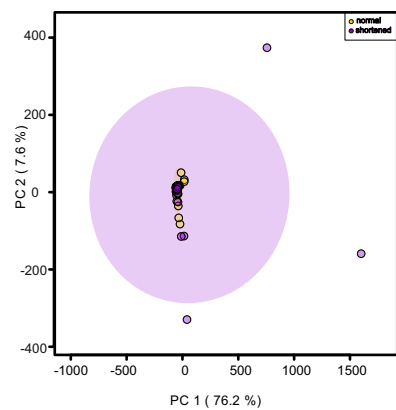**B**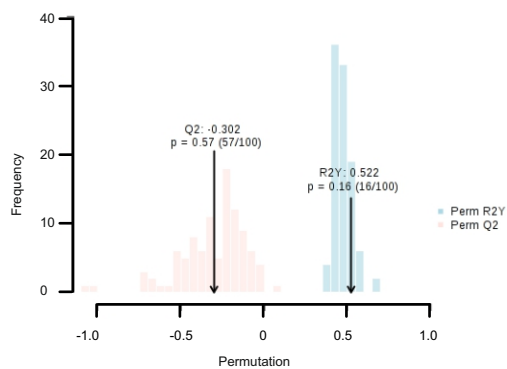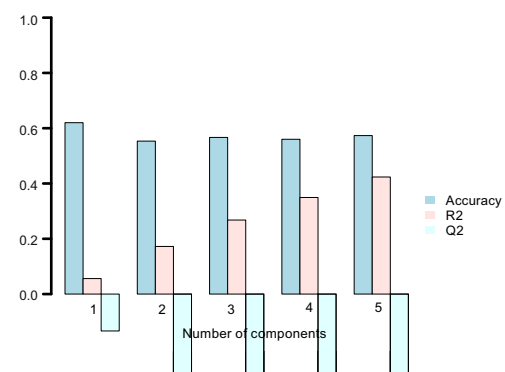**C**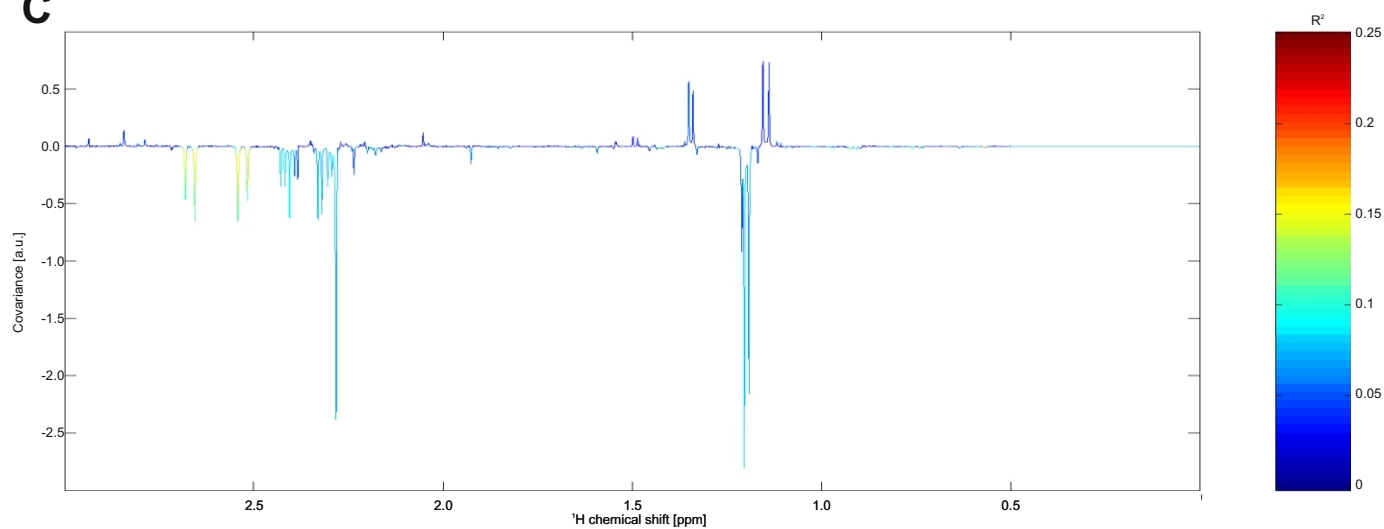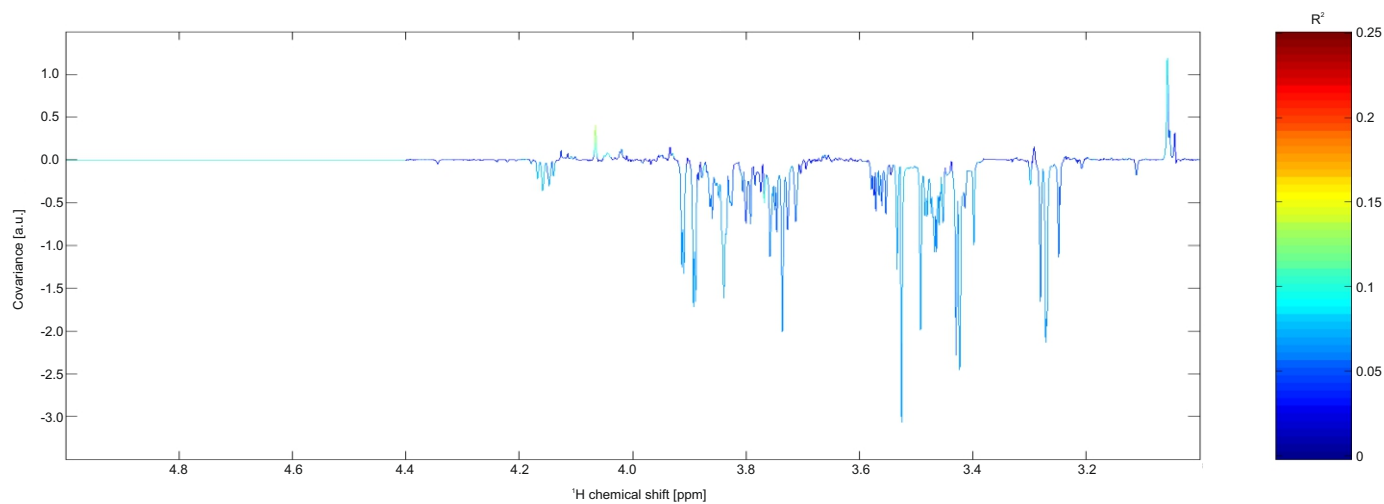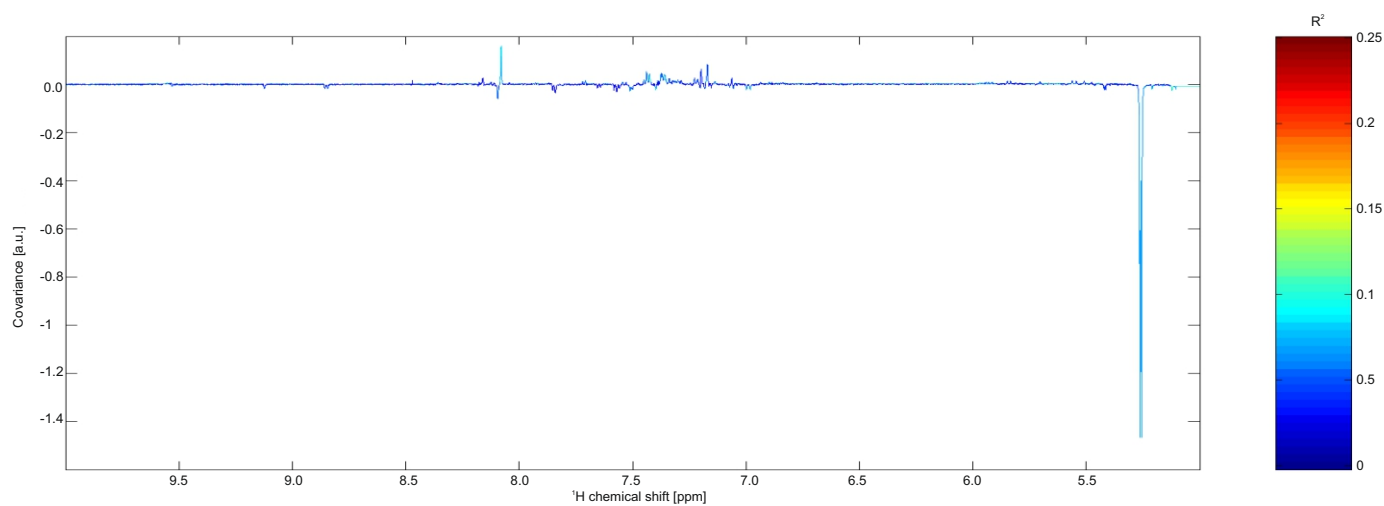

Supplement: FIG S5 [file mSystems.00334-20-sf005.pdf]

**A**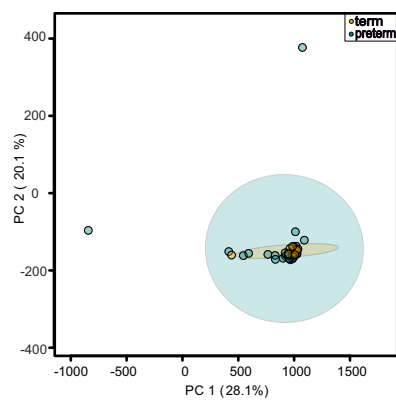**B**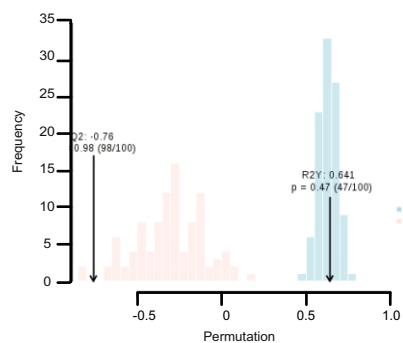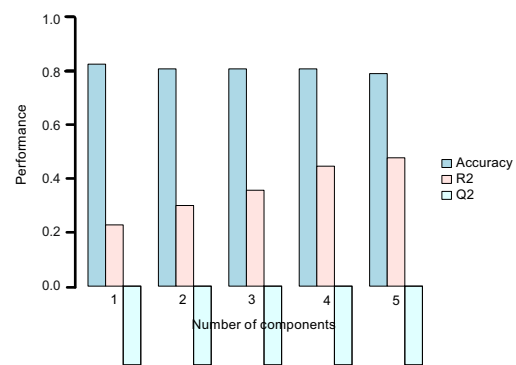**C**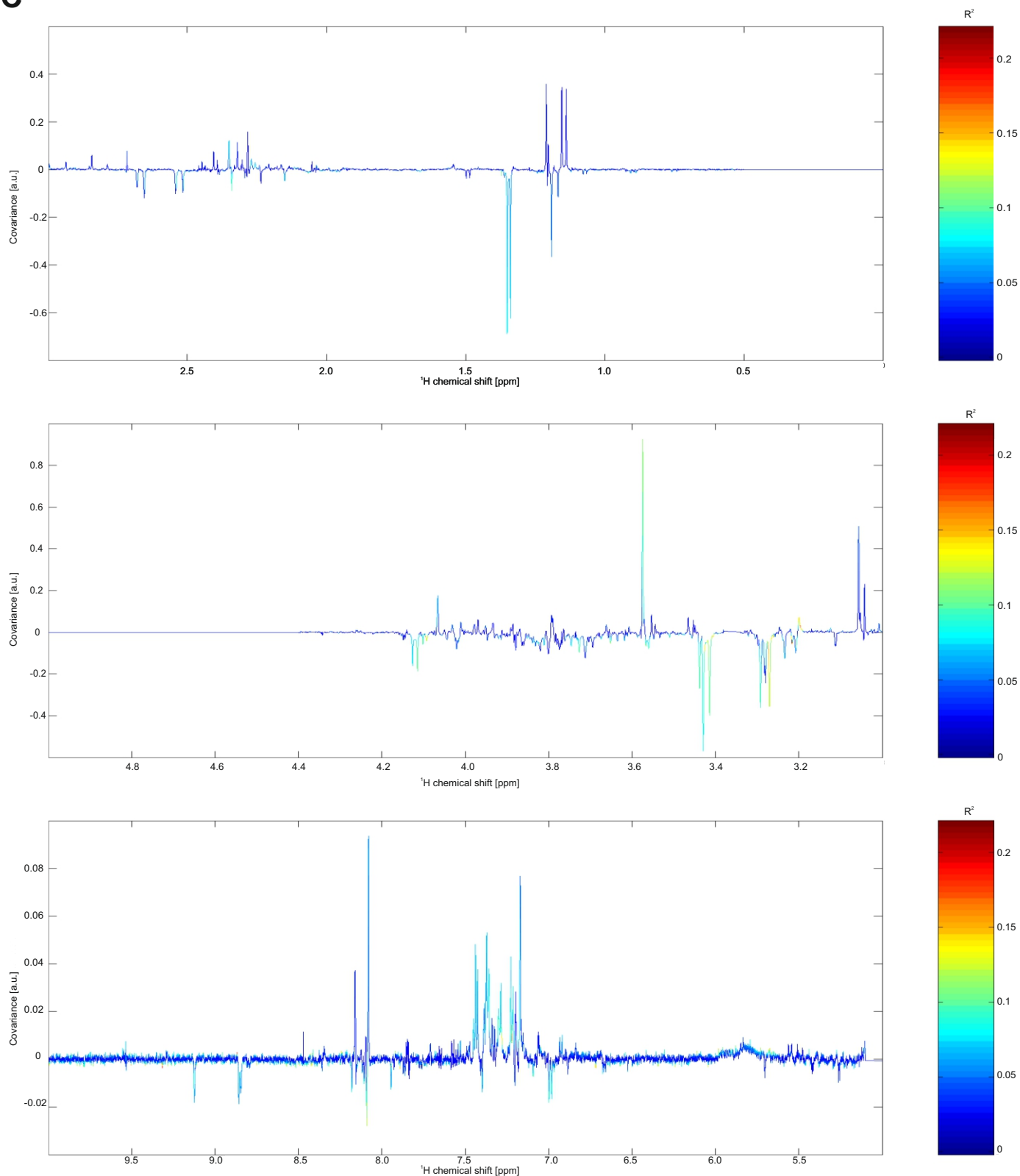

Supplement: FIG S6 [file mSystems.00334-20-sf006.pdf]

**A**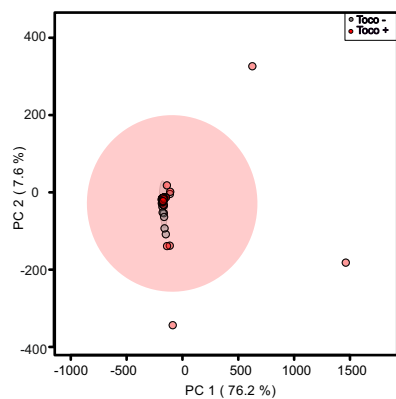**B**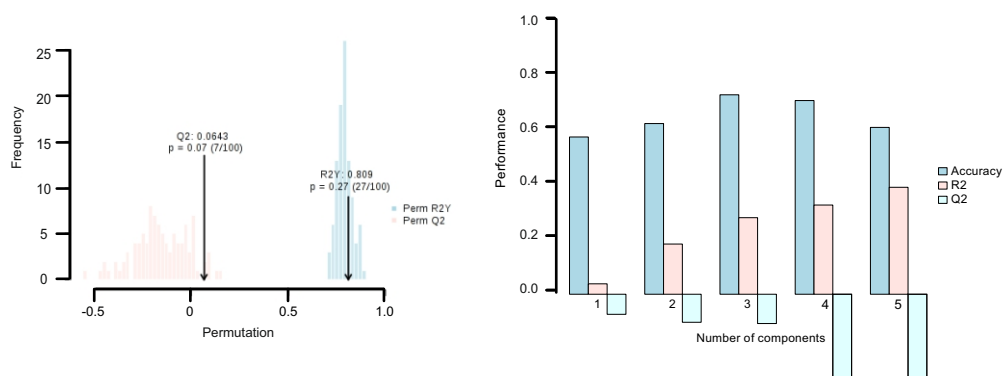**C**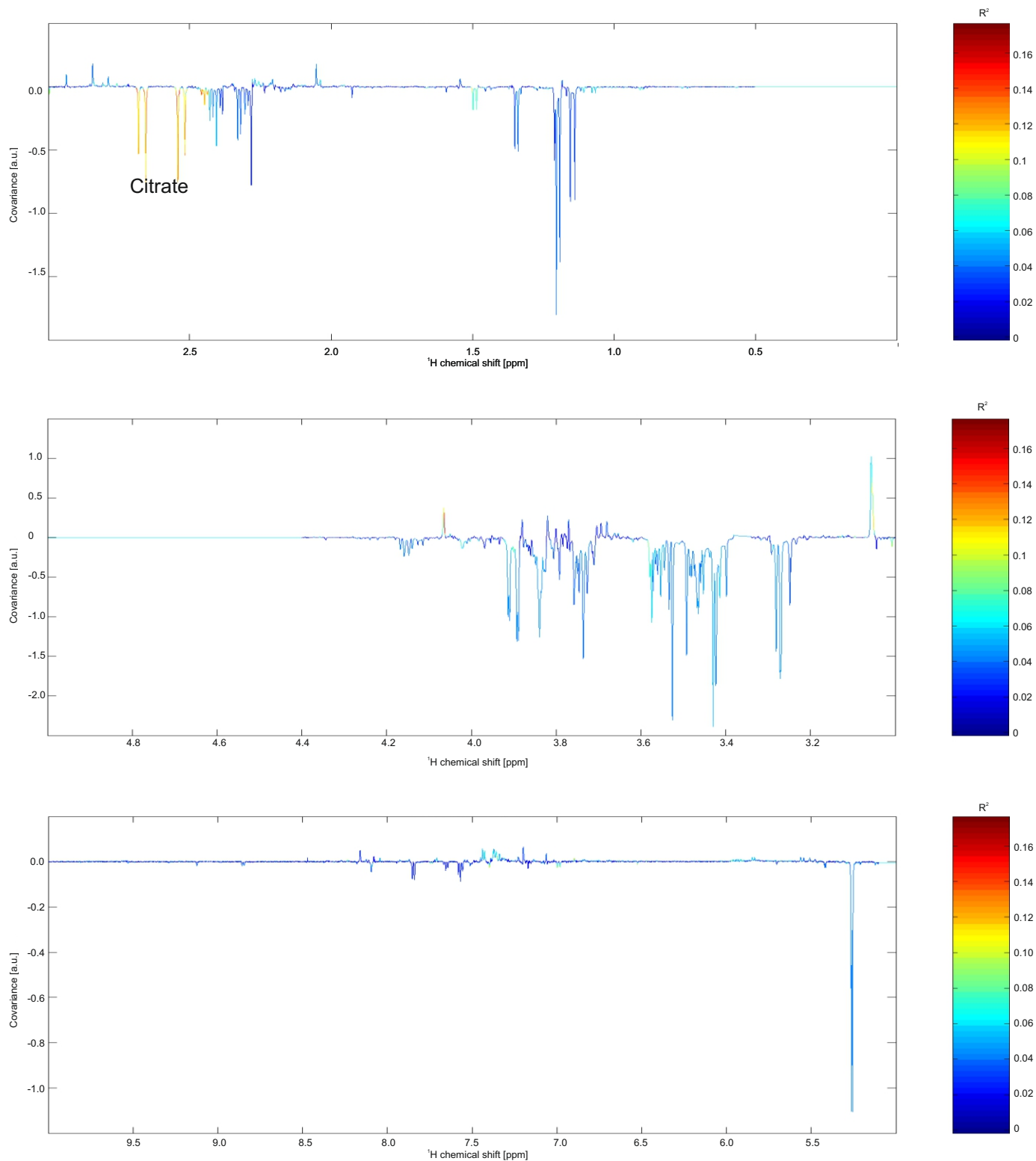

Supplement: FIG S7 [file mSystems.00334-20-sf007.pdf]
