# Supplementary material for: Human Milk Oligosaccharides Modulate the Risk for Preterm Birth in a Microbiome-Dependent and -Independent Manner
Source: mSystems. 2020 Jun 9;5(3):e00334-20. doi: 10.1128/mSystems.00334-20 (PMC7289590; doi:10.1128/mSystems.00334-20)
Supplement: FIG S8 [file mSystems.00334-20-sf008.pdf]

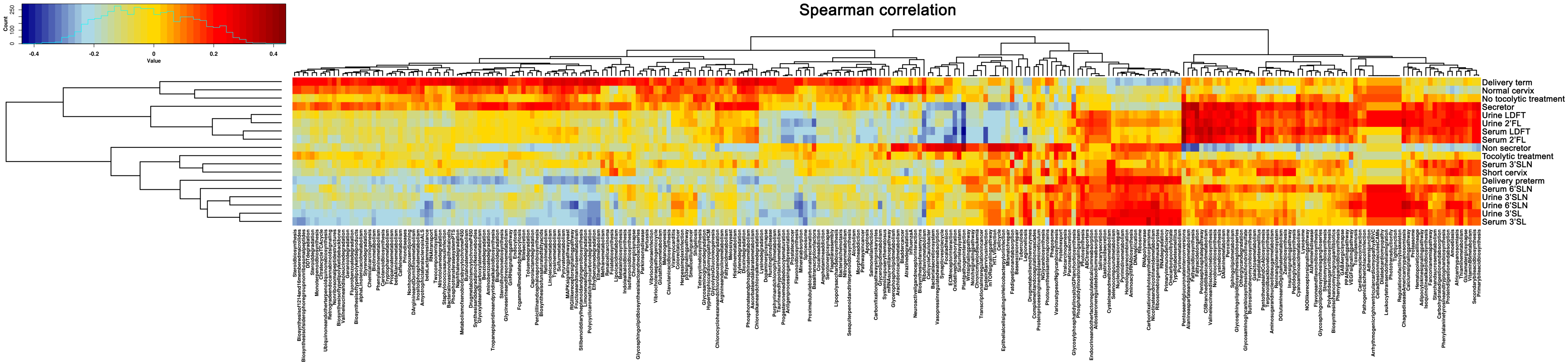
Spearman correlation
Count
Value
-0.4
-0.2
0
0.2
0.4
Delivery term
Normal cervix
No tocolytic treatment
Secretor
Urine LDFT
Urine 2'FL
Serum LDFT
Serum 2'FL
Non secretor
Tocolytic treatment
Serum 3'SLN
Short cervix
Delivery preterm
Serum 6'SLN
Urine 3'SLN
Urine 6'SLN
Urine 3'SL
Serum 3'SL
